# Artificial Intelligent‐Enhanced Metabolite Profiling for Intraoperative IDH1 Genotyping in Glioma Using an Orthogonally Responsive SERS Probe

**DOI:** 10.1002/advs.202503360

**Published:** 2025-04-02

**Authors:** Hang Yin, Xin Zhang, Zheng Zhao, Chong Cao, Minhua Xu, Suhongrui Zhou, Tian Xuan, Ziyi Jin, Limei Han, Yang Fan, Cong Wang, Xiao Zhu, Ying Mao, Jinhua Yu, Cong Li

**Affiliations:** ^1^ MOE Key Laboratory of Smart Drug Delivery MOE Innovative Center for New Drug Development of Immune Inflammatory Diseases School of Pharmacy Fudan University Shanghai 201203 China; ^2^ Department of Neurosurgery Huashan Hospital Shanghai Medical College Fudan University National Center for Neurological Disorders Shanghai Key Laboratory of Brain Function and Restoration and Neural Regeneration Neurosurgical Institute of Fudan University Shanghai Clinical Medical Center of Neurosurgery Shanghai 200040 China; ^3^ School of Information Science and Technology Fudan University Shanghai 200433 China; ^4^ Department of Ophthalmology Shanghai Key Laboratory of Orbital Diseases and Ocular Oncology Shanghai Ninth People's Hospital Shanghai Jiaotong University School of Medicine Shanghai 200011 China; ^5^ Artificial Intelligence Innovation and Incubation Institute Fudan University Shanghai 200433 China; ^6^ Zhongshan Hospital State Key Laboratory of Medical Neurobiology and MOE Frontiers Center for Brain Science Fudan University Shanghai 200032 China; ^7^ Greater Bay Area Institute of Precision Medicine Fudan University Guangzhou 511462 China

**Keywords:** glioma, gold nanoprobes, intraoperative IDH genotyping, molecular imaging, surface‐enhanced Raman scattering (SERS)

## Abstract

Intraoperative identification of the isocitrate dehydrogenase type 1 (IDH1) genotype, a key molecular marker in glioma, is essential for optimizing surgical strategies and tailoring post‐surgical treatments. However, current clinical practices lack effective methods for real‐time IDH1 genotype detection during surgery. Here, a novel strategy is proposed for intraoperative IDH1 genotype identification by simultaneously measuring two redox‐related metabolites. A surface‐enhanced Raman scattering (SERS) probe is developed to detect glutathione and hydrogen peroxide concentrations through orthogonally responsive Raman signals. Additionally, a deep learning algorithm is implemented, leveraging 2D Raman spectra transformation and multi‐task learning to enhance measurement speed and accuracy. This AI‐assisted SERS approach can identify the IDH1 genotype in glioma patients within 7 min. In a cohort of 31 glioma patients, the system achieved an area under the receiver operating characteristic curve of 0.985 for accurate IDH1 genotype differentiation. This study holds significant promise for refining surgical decision‐making and personalizing post‐surgical treatments by enabling rapid intra‐operative molecular biomarker identification.

## Graphical abstract

This study presents a novel approach for intraoperative identification of the IDH1 genotype in glioma patients using a surface‐enhanced Raman scattering (SERS) probe to measure glutathione and hydrogen peroxide. By integrating AI‐driven deep learning algorithms, the method achieves rapid and accurate genotype differentiation, offering the potential to optimize surgical strategies and tailor post‐surgical treatments.

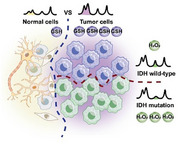

## 1 Introduction

Surgery remains the cornerstone for treatment of glioma, the most common malignant brain tumor.^[^ ^1^ ^]^ Due to their highly infiltrative nature, surgeons frequently encounter challenges in defining the invasive margins of gliomas.^[^ ^2^ ^]^ Incomplete excision typically leads to rapid relapse, while aggressive resection risks damaging non‐regenerative nerves, vessels, or the eloquent cortex. Although a higher extent of resection generally correlates with improved prognosis, outcomes vary among glioma patients with different genomic subtypes. The recent fifth edition of the World Health Organization (WHO) classification of gliomas, published in 2021, integrates histopathology with molecular characteristics,^[^ ^3^ ^]^ highlighting the pivotal role of molecular features in glioma grading. Among these, isocitrate dehydrogenase type 1 (IDH1) stands out as a critical molecular marker for categorizing gliomas. In contrast to a median overall survival of 34.6 months for IDH1‐wildtype (IDH1‐WT) patients with histologically low‐grade glioma (LGG), this value was determined to be 76.0 months for IDH1‐mutant (IDH1‐MUT) LGG patients.^[^ ^4^ ^]^ Despite the overarching goal of maximal safe resection, surgical approaches differ significantly between IDH1 subtypes. For IDH1‐WT glioma, the strategy focuses on achieving the maximum safe resection due to their poor response to chemotherapy and radiotherapy.^[^ ^5^ ^]^ Conversely, for IDH1‐MUT gliomas, the priority shifts toward preserving the functional brain tissues, reflecting their sensitivity to chemotherapy and radiotherapy.^[^ ^6^ ^]^ Therefore, intraoperative IDH1 genotyping of glioma enables physicians to promptly optimize surgical strategies and optimizing postoperative treatments.

The current gold standard for detecting IDH1 mutations, including immunohistochemistry and genomic sequencing, is impractical for intraoperative use due to lengthy processing times. As an emerging method for detecting unique signals from the molecules of interest, magnetic resonance spectroscopy (MRS)^[^ ^7^ ^]^ identifies IDH1 mutation by tracking D‐2‐hydroxyglutarate (D‐2HG), an oncometabolite of IDH1‐MUT glioma cells. However, D‐2HG MRS has not been widely used due to challenges in optimizing data acquisition sequences and the lack of commercial sequences or post‐processing software.^[^ ^8^ ^]^ Mass spectrometry (MS) techniques, such as desorption electrospray ionization‐mass spectrometry,^[^ ^9^ ^]^ gas chromatography‐mass spectrometry,^[^ ^10^ ^]^ and direct capillary spray^[^ ^11^ ^]^ showed feasibility in intraoperatively identifying IDH1 genotypes with simplified sample preparation procedures. However, their limitations include high equipment costs, difficulty distinguishing D‐2HG from its chiral enantiomer, and inability to spatially and quantitatively access the IDH1 mutational heterogenicity in glioma samples. Consequently, there is an urgent need to develop new techniques for intraoperative identification of IDH1 genotypes.

As an indispensable enzyme, IDH1 is involved in multiple metabolic processes, including the Krebs cycle, glutamine metabolism, lipogenesis, and redox regulation.^[^ ^12^ ^]^ IDH1 catalyzes the conversion of isocitrate to α‐ketoglutarate (α‐KG), thereby producing essential reduced nicotinamide adenine dinucleotide phosphate (NADPH) for the regeneration of reduced glutathione (GSH), a key scavenger of cellular reactive oxygen species (ROS). The IDH1 mutation fundamentally impedes the generation of NADPH and α‐KG, compromising ROS‐scavenging capacity and ultimately leading to intracellular hydrogen peroxide (H_2_O_2_) accumulation.^[^ ^13^ ^]^ Consequently, gliomas harboring IDH1‐MUT exhibit higher H_2_O_2_ levels than IDH1‐WT counterparts, making H_2_O_2_ a potential diagnostic biomarker for detecting IDH1 mutations.^[^ ^14^ ^]^ Meanwhile, the intracellular GSH is reduced by the consumption of excess ROS in IDH1‐MUT glioma. Actually, the tumoral GSH levels were observed negatively correlated with the D‐2HG concentration.^[^ ^15^ ^]^ Therefore, we propose a strategy for intraoperative determination of glioma IDH1 genotype by simultaneously determining the intracellular concentrations of both H_2_O_2_ and GSH.

Currently, the available techniques for determining redox‐associated metabolites include fluorescence imaging and assay kits. Multiple metabolite detection using fluorescence imaging typically requires the administration of several fluorescence probes. However, their cross‐talking fluorescence signals and inconsistent pharmacokinetics compromise measurement accuracy. On the other hand, metabolite assay kits involve complex procedures and lengthy operation times. Additionally, they provide information limited to a specific region at a specific time point, lacking temporal and spatial resolution. Surface‐enhanced Raman scattering (SERS) has emerged as a powerful technique that enhances the Raman signal of molecules when they interact with specific nanostructured materials.^[^ ^16^ ^]^ SERS offers advantages such as high sensitivity, a non‐destructive nature, and multiplexing capabilities, making it particularly suitable for the concurrent detection of multiple metabolites.^[^ ^17^ ^]^ For instance, Tian et al. developed a SERS optophysiological nanoneedle for simultaneous sensing both CO_3_^2−^ and pH in a live mouse brain.^[^ ^18^ ^]^ In addition, we developed pH‐responsive SERS probes to delineate the invasive margins of gliomas.^[^ ^19^ ^]^ Hence, a SERS probe capable of simultaneously determining intracellular GSH and H_2_O_2_ holds promise in the identification of IDH1 genotypes of glioma cells.

**Scheme 1:**
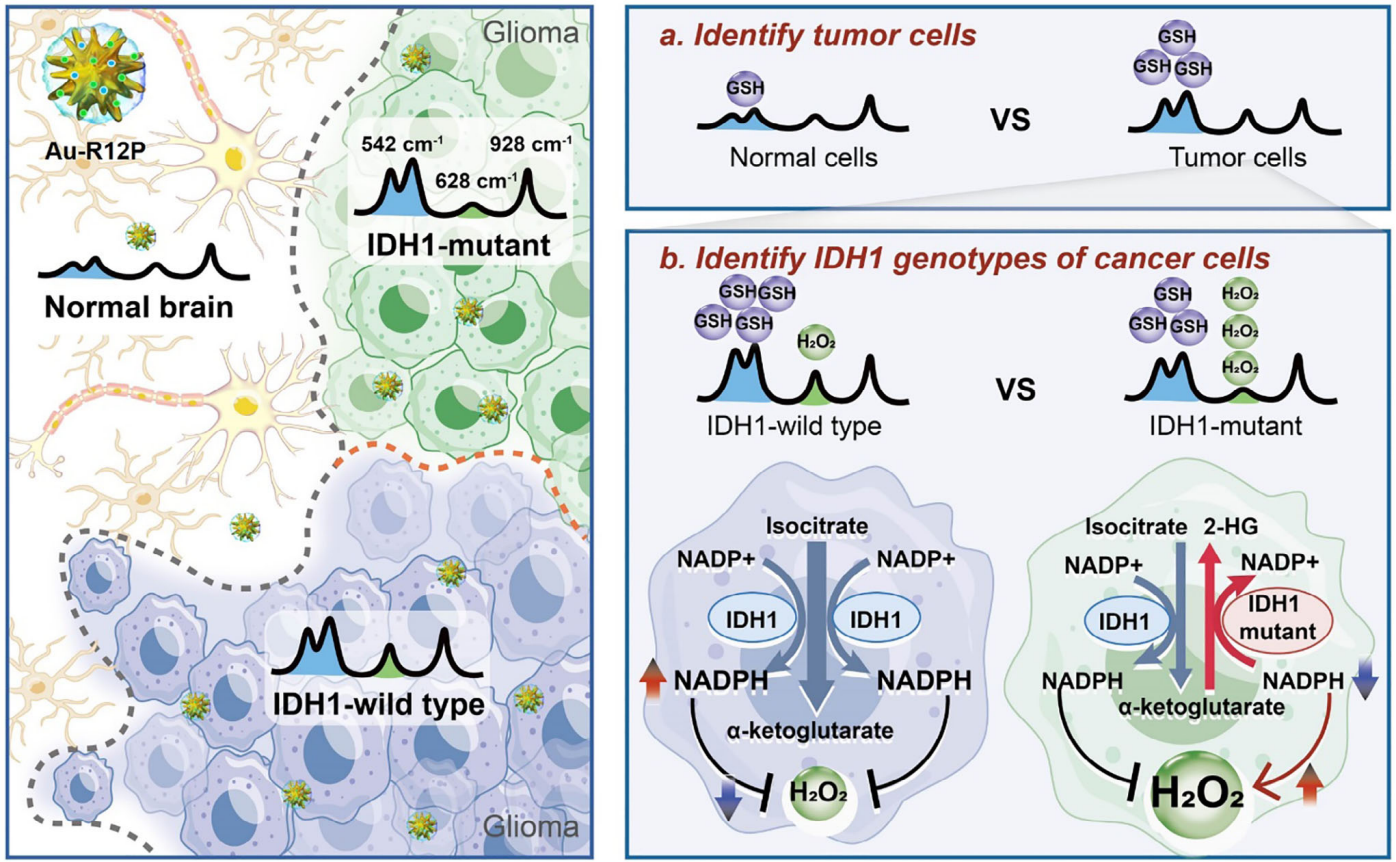
Identifying IDH1 genotypes of gliomas by determining redox‐associated metabolites. A SERS nanosensor, Au‐R12P, was developed for simultaneously determining the intracellular concentrations of GSH and H_2_O_2_ via ratiometric Raman signals. High intracellular concentration of GSH in glioma cells triggers a remarkable enhancement of Raman peak of Au‐R12P at 542 cm^−1^, which helps to identify glioma tissue from normal brain tissue by measuring the Raman intensity ratio of I_542_/I_928_. Subsequently, the increased H_2_O_2_ concentration but concomitantly decreased GSH concentration in IDH mutated glioma cells leads to a reduction of the I_628_/I_542_ ratio, which aids surgeon to differentiate IDH genotypes of glioma intraoperatively.

In this study, we introduce a SERS‐based approach coupled with a deep learning model to intraoperatively determine IDH1 status in glioma patients by determining GSH and H_2_O_2_ concentrations on freshly excised tumor tissue. This strategy not only aims to enhance the precision of glioma surgeries but also aligns with the broader goal of personalized surgical interventions, potentially improving patient prognoses(**Scheme** **1**).

## 2 Results

### 2.1 Synthesis and Characterization of SERS Probe Au‐R12P

To simultaneously determine multiple redox‐associated metabolites, the SERS probe must meet specific criteria. First, to avoid cross‐interference, the Raman reporters sensing GSH or H_2_O_2_ should exhibit non‐overlapping Raman spectra. Second, the SERS probe must show orthogonal specificity to GSH and H_2_O_2_ without interference from other endogenous ions or metabolites. Third, a high response velocity is needed considering the limited operation time window. Lastly, the incorporation of a ratiometric strategy is crucial for the quantitative determination of the target metabolites.

**Figure 1:**
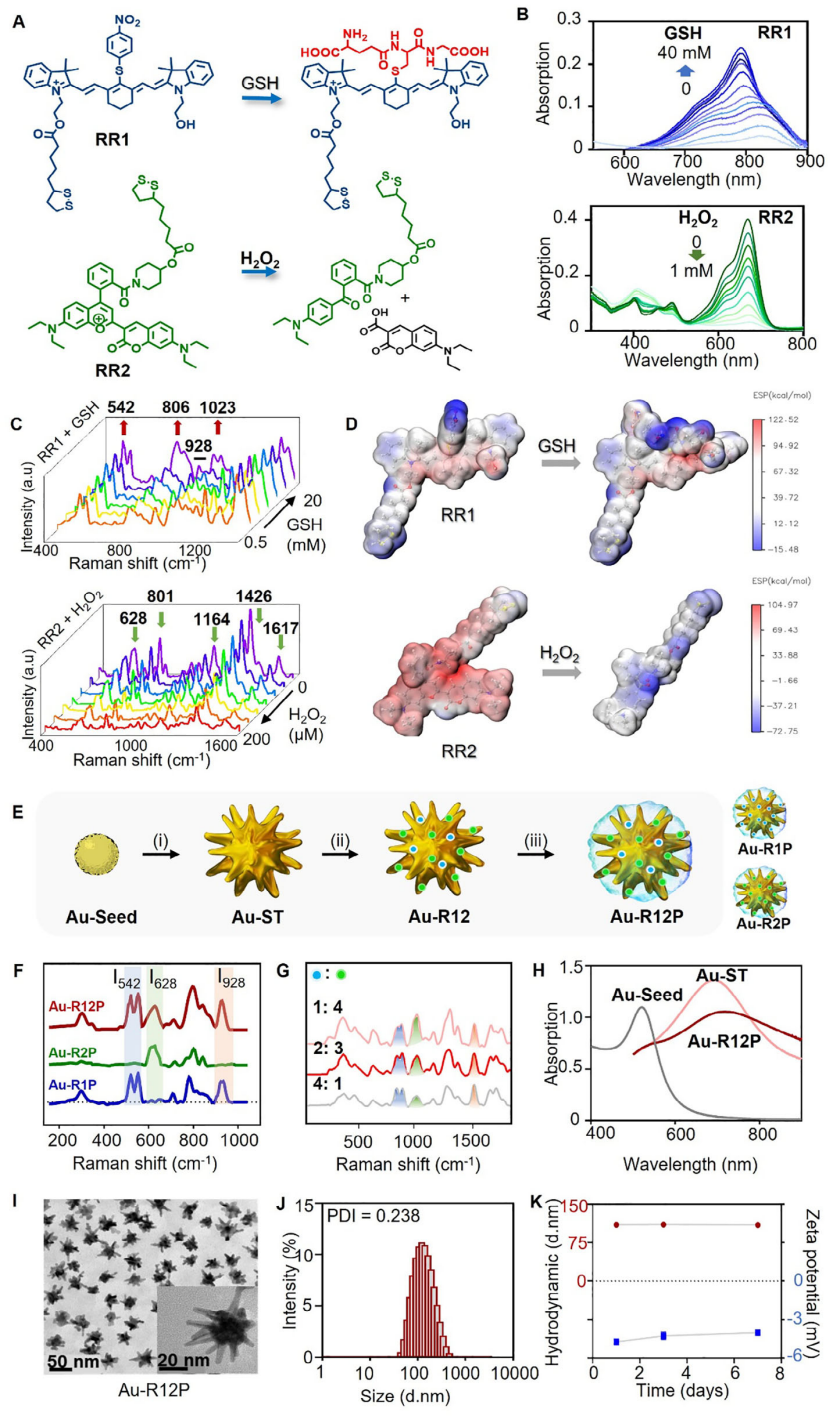
Synthesis and characterization of SERS probe Au‐R12P. A) Chemical structures of Raman molecular reporters RR1 and RR2, which respond to GSH and H_2_O_2_, respectively. B) Absorbance of RR1 (5 × 10^−6^ m, upper panel) and RR2 (1 × 10^−5^ m, lower panel) as a function of GSH and H_2_O_2_ concentration, respectively. C) GSH‐dependent SERS spectra of RR1 and H_2_O_2_‐dependent SERS spectra of RR2. GSH: 0.5‒20 mm. H_2_O_2_: 0‒200 µm. D) Electrostatic potential (ESP) map of Raman reporter molecule RR1 and RR2 before and after the treatment of GSH and H_2_O_2_, respectively. E) Synthetic procedure of Au‐R12P. (i) Ascorbic acid, AgNO_3_, 40 s, room temperature (r.t.); (ii) RR1, RR2, 1.0 h, r.t.; (iii) HS‐PEG^5k^‐OCH_3_, 12 h, r.t. In step (ii), when the reporter molecules added were entirely RR1 or entirely RR2, the control probes Au‐R1P or Au‐R2P were obtained. F) Raman spectra of Au‐R12P and control probes Au‐R1P, Au‐R2P after excitation at 785 nm. The GSH response peak (I_542_), H_2_O_2_ response peak (I_628_), and reference peak (I_928_) can be separated well from each other in the Raman spectrum of Au‐R12P. G) Raman spectra of Au‐R12P as a function of molar ratio between Raman reporters RR1 and RR2 modified on nanoparticle surface (RR1:RR2 = 1:4, 2:3, 4:1). H) Absorption of gold seeds, gold nanostars, and Au‐R12P in aqueous solution. I) TEM images of Au‐R12P. Scale bar: 50 nm. Inset: locally amplified image. Scale bar: 20 nm. J) Size distribution and PDI variation of Au‐R12P. K) Hydrodynamic diameter and ζ‐potential of Au‐R12P as a function of incubation time in aqueous solution.

Considering the above perquisitions, we first synthesized two Raman reporters (RR) with distinct structural skeletons, designated as RR1 and RR2 (**Figure** **1**A). Notably, RR1 and RR2 exhibited concentration‐dependent responses to GSH and H_2_O_2_, respectively (Figure 1B). The double Raman peak at 542 cm⁻¹, peaks at 806 and 1023 cm⁻¹ of RR1 exhibited increased enhancement with rising GSH concentration (Figure 1C), corresponding to the C‐C out‐of‐plane bending of indolenium rings (542 cm⁻¹), C‐C stretching vibrations of the conjugated chain (806 cm⁻¹), and CH_2_ bending vibrations (1023 cm⁻¹) in the π‐conjugated unit.^[^ ^20^ ^]^ The peak at 928 cm⁻¹, associated with non‐conjugated units (C‐C‐C bending and CH_2_ twisting), remained unchanged in intensity and served as a reference peak. Replacing the nitrobenzene group in RR1 with electron‐donating GSH increased the electron cloud density of the indole ring system (Figure 1D), enhancing the polarizability of the molecule and boosting the Raman scattering signal, particularly at 542, 806, and 1023 cm⁻¹. Almost all Raman peaks of RR2, including those at 628, 801, 1164, 1426, and 1617 cm⁻¹, were significantly reduced as the H₂O₂ concentration increased (Figure 1C,D). This decrease was attributed to the disruption of RR2's large conjugated structure, leading to a significant reduction in its electron cloud density. When the Raman spectra of the two Raman reporters were combined, the peaks at I_542_, I_628_, and I_928_ remained distinguishable, but other peaks overlapped and interfered with each other's response (Figure S1, Supporting Information).

RR1 and RR2 were then conjugated onto the surface of gold nanostar (Au‐ST), and further coated with polyethylene glycol (MW: 5 kDa, PEG^5k^) to generate the aiming SERS probe Au‐R12P (Figure 1E; Figure S2, Supporting Information). The GSH responsive Raman peak (I_542_) and the H_2_O_2_ responsive peak (I_628_), along with the self‐calibrating reference peak (I_928_) were observed separately on the Raman spectrum of Au‐R12P (Figure 1F). We further optimized the molar ratio between the reporter molecules, finding that a 2:3 ratio of RR1 to RR2 provided the optimal dual metabolite‐responsive performance (Figure 1G). The absorbance of nanoparticles ranged from 660 to 800 nm with a maximum at 710 nm (Figure 1H). Notably, the energy to an electronic transition of the Raman reporters matched the incident photon energy, enhancing signal intensity and sensitivity through the surface‐enhanced resonance Raman scattering (SERRS) effect.^[^ ^21^ ^]^ Transmission electron microscopy (TEM) images showed Au‐R12P had a well‐dispersed morphology with an average diameter of 60 nm (Figure 1I). Dynamic light scattering (DLS) determined the average hydrodynamic diameter of Au‐R12P was ≈110 nm and the Zeta potential was −4.7 mV. Notably, Au‐R12P demonstrated good colloidal stability, maintaining consistent hydrodynamic diameter and Zeta potential in aqueous solution even after one week (Figure 1J,K). Furthermore, it reliably detected concentrations as low as 50 pm, with no interference from background noise (Figure S3, Supporting Information). The probe also exhibited tissue penetration up to 0.5 mm, although signal intensity decreased significantly beyond this depth (Figure S4, Supporting Information).

### 2.2 Au‐R12P Orthogonally Determines GSH and H2O2 Concentrations

**Figure 2:**
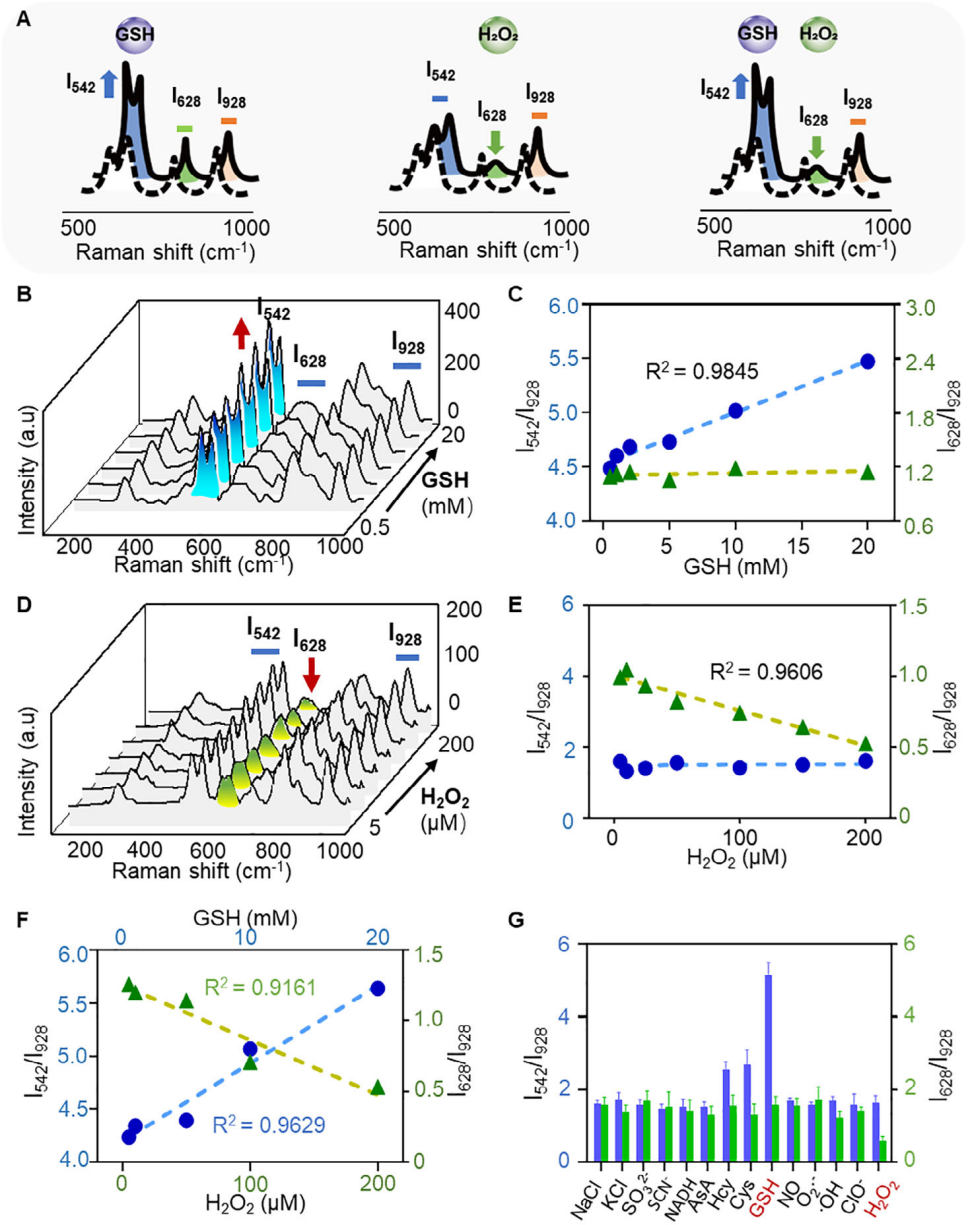
Au‐R12P enables simultaneous determination of GSH and H_2_O_2_. A) Schematic representation of the ratiometrically responsive characteristics of Au‐R12P to redox‐associated metabolites. B) Raman spectra of Au‐R12P in PBS as a function of GSH concentrations (0.5‒20 mm) in physiologic range. C) Plots of the Raman intensity ratios (I_542_/I_928_ and I_628_/I_928_) of Au‐R12P as a function of GSH concentrations (GSH increased from 0.5 to 20 mm; H_2_O_2_ = 0 mm), (*n* = 5). D) Raman spectra of Au‐R12P in PBS as a function of H_2_O_2_ concentration in physiologic range (5‒200 µm). E) Plots of Raman intensity ratio (I_542_/I_928_ and I_628_/I_928_) as a concentration of H_2_O_2_ (H_2_O_2_: 5‒200 µm; GSH = 0 mm), (*n* = 5). F) GSH and H_2_O_2_ concentration‐dependent Raman signal intensity ratios (I_542_/I_928_ and I_628_/I_928_) of Au‐R12P (*n* = 5) in an aqueous solution comprising both H_2_O_2_ (1, 10, 50, 100, and 200 µm) and GSH (0.1, 1, 5, 10, 20 mm). G) I_542_/I_928_ and I_628_/I_928_ values of Au‐R12P (100 × 10^−9^ m) in the presence of selected endogenous ions or metabolites for two hours. (NaCl, KCl, SO_3_^2−^, SCN^−^, NADH, AsA, Cys, Hcy, Cys, GSH: 10 mm; NO, O_2_∙^−^, ∙OH, ClO^−^, H_2_O_2_: 100 µm). AsA, ascorbic acid; Hcy, homocysteine; Cys, cysteine.

**Figure** **2**A presents the ratiometric response of Au‐R12P to varying concentrations of GSH and H_2_O_2_. The Raman peak at 542 cm^−1^ (I_542_) increases as a function of GSH concentration, while the characteristic peak at 628 cm^−1^ (I_628_) decreases in response to H_2_O_2_. Furthermore, the Raman peak intensity at 928 cm^−1^ (I_928_) remains constant, serving as an internal reference. As shown in Figure 2B, there is a consistent enhancement in the intensity of I_542_ with increasing GSH concentrations, whereas the intensities of I_628_ and I_928_ remain unchanged. Consequently, the Raman signal intensity ratio of I_542_/I_928_ displayed a robust linear positive correlation with the GSH concentration, while the I_628_/I_928_ ratio showed minimal variation (Figure 2C). An increase in H_2_O_2_ concentration resulted in a discernible decrease in the I_628_ intensity, with I_542_ and I_928_ intensities remaining consistent (Figure 2D,E). Significantly, Au‐R12P enabled the concurrent quantification of GSH and H_2_O_2_ concentrations in their mixed solution without mutual interference (Figure 2F; Figure S5, Supporting Information). Additionally, the Raman intensity ratios of I_542_/I_928_ and I_628_/I_928_ were minimally affected by the presence of physiological ions, such as Na^+^, K^+^, and Cl^−^, reductants (SO_3_^2−^, SCN^−^, NADH, ascorbic acid (AsA), cysteine (Cys), homocysteine (Hcy)), and biologically related redox chemicals (NO, O_2_·^−^, **·**OH, ClO^−^), confirming the high specificity of Au‐R12P to GSH and H_2_O_2_ (Figure 2G). These studies demonstrate that Au‐R12P can specifically and quantitatively identify H_2_O_2_ and GSH.

### 2.3 Development of Deep Learning Model Processing Raman Spectra

**Figure 3:**
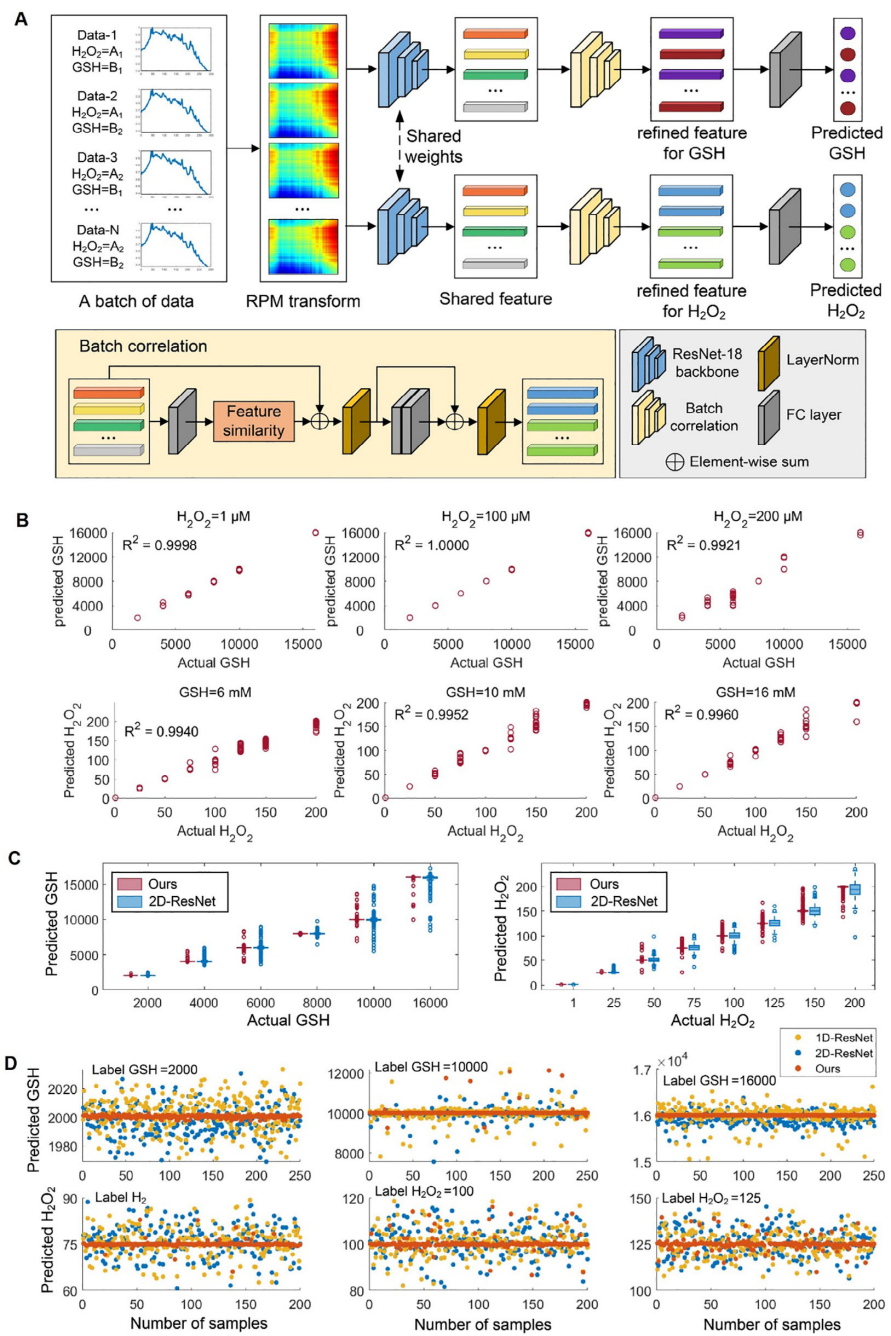
Deep learning model DBCNet simultaneously determining GSH and H_2_O_2_. A) Schematic diagram of the proposed DBCNet for analyzing the Raman spectra of Au‐R12P in presence of redox‐associated metabolites. Given a mini‐batch consisting of *N* randomly chosen Raman spectra of Au‐R12P, the spectra in the batch are converted into 2D images by Relative Position Matrix (RPM), and their feature vectors are initialized using a ResNet‐18 backbone. Then, a dual‐branch structure is constructed to separately refine the initial features of GSH (or H_2_O_2_) by batch correlation. In batch correlation, the feature similarity between pairwise spectra in the batch is calculated. Finally, the fully‐connected layers are applied to obtain the predicted concentrations of GSH and H_2_O_2_. B) Linear regression plots showing the predicted concentrations of GSH (or H_2_O_2_) in presence of three different concentrations of H_2_O_2_ (or GSH) using DBCNet. C) Boxplot comparison of predicted GSH and H_2_O_2_ concentrations between 2D‐ResNet and DBCNet. D) Scatterplot comparison of predicted GSH and H_2_O_2_ concentrations among 1D‐ResNet, 2D‐ResNet and DBCNet.

The existing deep learning methods for Raman spectroscopy, whether 1D convolutional neural networks (1D‐CNNs) or 2D‐CNNs,^[^ ^22^ ^]^ only focus on intra‐spectra feature learning, ignoring the inter‐spectral feature differences. In this work, we propose a 2D dual‐branch batch correlation network (DBCNet) for determining the concentrations of GSH and H_2_O_2_ by analyzing Raman spectra (**Figure** **3**A). First, the spectra were converted into the Relative Position Matrix (RPM)^[^ ^23^ ^]^ as Equation (1).

(1) $$RPM_{i,j}=x_{i}\;-x_{j},i,j=1,2,\text{…},M$$

where *M* is the length of the spectrum, and *x_i_* is the *ith* point of the spectrum. The spectral images were then fed into ResNet‐18 backbone to extract the shared feature for GSH and H_2_O_2_. On the basis, we constructed a dual‐branch architecture to decouple the feature learning of two metabolites for obtaining their respective refined features using batch correlation. In batch correlation, we measured the feature similarity between pairwise spectra in a batch and update the features for GSH (or H_2_O_2_) as Equation (2).

(2)

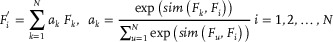

where *N* is the size of batch, *F_k_* is the original *kth* feature in the batch, $F_{i}^{\prime }$ is the updated *ith* feature in the batch, *a_k_* is the weight that *F_k_* contributes to *F_i_*, *sim*(·) is cosine similarity. Finally, a fully connected layer outputs the predicted concentration of GSH and H_2_O_2_. We build a database containing 9600 Raman spectra with diverse concentrations of GSH and H_2_O_2_. 20% of the data was randomly selected as the test set, and the remaining data was divided into the training set and the validation set at the ratio of 9:1. The linear regression plots of the predicted GSH (H_2_O_2_) concentrations with DBCNet are shown in Figure 3B. The predicted concentrations of GSH and H_2_O_2_ exhibited high accuracy, with a coefficient of determination (R^2^) above 0.99. Moreover, the predicted performance of DBCNet surpassed that of the two commonly used models (1D‐ResNet and 2D‐ResNet),^[^ ^24^ ^]^ in almost all scenarios (Figures S6 and S7, Supporting Information). The boxplot comparison of the predicted concentrations of GSH and H_2_O_2_ between 2D‐ResNet and DBCNet are shown in Figure 3C. Notably, DBCNet outperforms 2D‐ResNet in prediction accuracy, with a narrower interquartile range. Additionally, we randomly selected 250 spectra under three different GSH concentrations and 200 spectra under three different H_2_O_2_ concentrations for scatterplot comparison (Figure 3D). The prediction results from our model showed superior stability around the ground truth (Figure S8, Supporting Information). Tables S1 and S2 (Supporting Information) provided a comprehensive summary of the concentration prediction results of GSH and H_2_O_2_ with 1D‐ResNet, 2D‐ResNet and DBCNet on three key performance indexes, the mean absolute error (MAE), standard deviation of absolute error (SD) and root mean squared error (RMSE). Compared with the alternative models, DBCNet exhibited an average enhancement of 63%, 31%, and 33% for GSH prediction, and 66%, 20%, and 32% for H_2_O_2_ prediction across the respective metrics, signifying the efficacy of this approach.

### 2.4 Au‐R12P Discriminates IDH Genotypes of Live Cancer Cells in Cell Cultures

**Figure 4:**
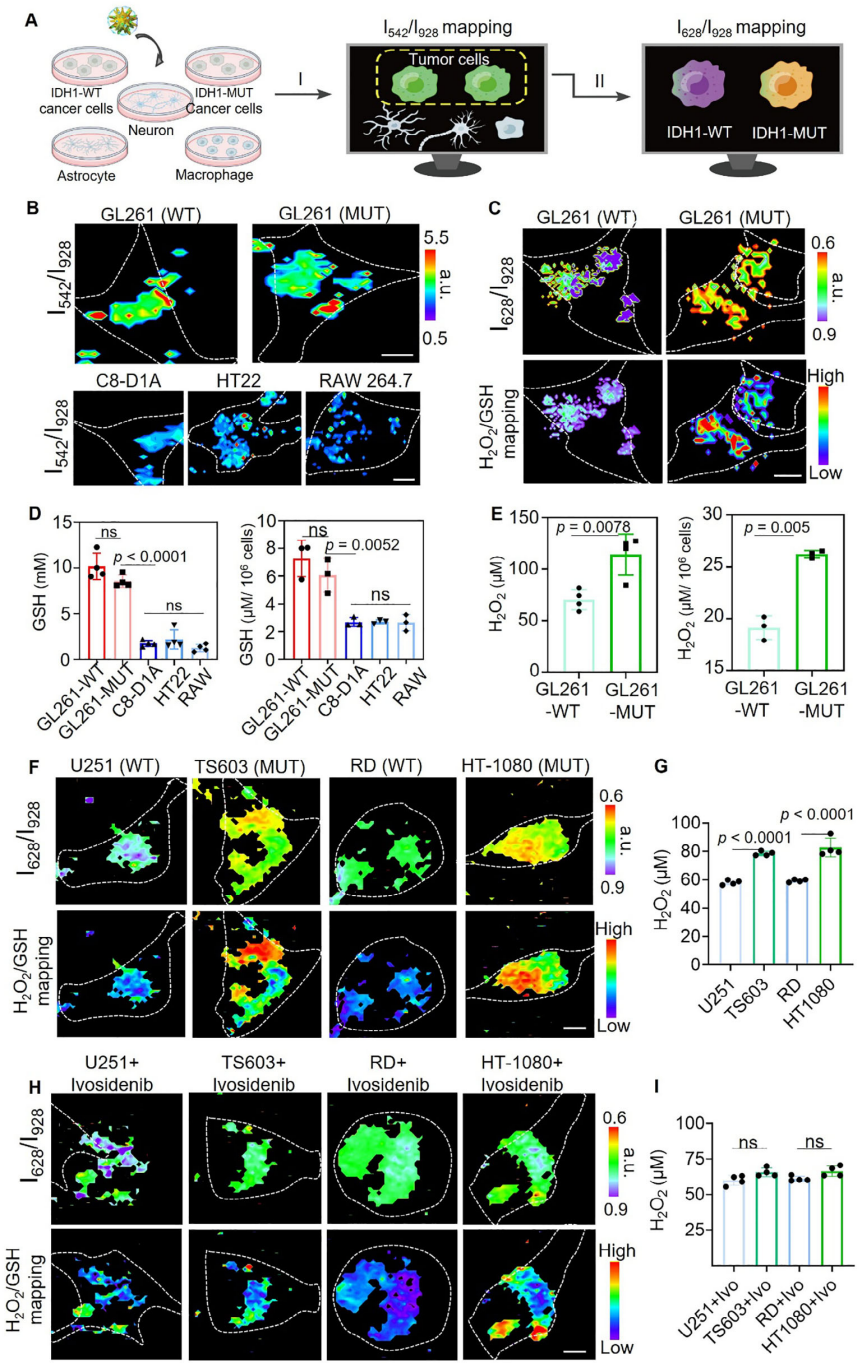
Au‐R12P identifies IDH1 genotypes of live cancer cells. A) Schematic workflow of identifying IDH1 genotype of live cells. The first step involves distinguishing tumor cells from normal brain cells based on the Raman intensity ratio I_542_/I_928_. The second step entails discriminating between IDH1‐WT and IDH1‐MUT cells based on the Raman ratio of I_628_/I_928_. B) Confocal Raman microscopic images of live GL261 (IDH1‐WT/MUT) glioma cells, C8‐D1A astrocyte, HT22 neuron, and RAW 264.7 macrophage cells after treatment with Au‐R12P (10 × 10^−8^ m) for 4 h. GSH concentration maps were generated by measuring the Raman signal intensity ratio (I_542_/I_928_). Scale bar: 10 µm. λ_ex_ = 785 nm. Cell margins are marked by white dotted lines. C) H_2_O_2_ concentration maps (upper) of live GL261 (IDH1‐WT/MUT) glioma cells generated by measuring the Raman peak ratio (I_628_/I_928_). The distribution map of H_2_O_2_/GSH ratio in tumor cells (lower). Scale bar: 10 µm. λ_ex_ = 785 nm. D) Average intracellular concentrations of GSH, as measured by Au‐R12P, in different types of live cells (left panel, *n* = 4). GSH levels in the above cells were measured by commercial kits (right panel, *n* = 3). E) Average intracellular concentrations of H_2_O_2_, as measured by Au‐R12P, in live GL261 (IDH1‐WT/MUT) glioma cells (left panel, *n* = 4). H_2_O_2_ levels in live GL261 (IDH1‐WT/MUT) glioma cells were measured by commercial kits (right panel, *n* = 3). F) H_2_O_2_ concentration maps (upper) of Au‐R12P in live U251 (WT), TS603 (MUT), RD (WT), and HT1080 (MUT) cells by measuring the Raman peak ratio (I_628_/I_928_). The distribution map of H_2_O_2_/GSH ratio (lower) in tumor cells. Scale bar = 9 µm. H) H_2_O_2_ concentration maps and H_2_O_2_/GSH maps of Au‐R12P in live U251, TS603, RD, and HT1080 cells following ivosidenib treatment. Scale bar = 9 µm. G,I) Average intracellular H_2_O_2_ concentrations measured by Au‐R12P in cells from panels F and H. The ordinary one‐way ANOVA is used for the multiple comparisons of data in panels D, G, and I. An unpaired *t*‐test is used to compare two sets of data in panel E. Statistical significance is determined for a *p*‐value of <0.05.

The capability of Au‐R12P to identify IDH genotypes was examined in cultured IDH1‐WT and IDH1‐MUT GL261 glioma cells. We selected 4 hours as the optimized incubation time since Au‐R12P uptake plateaued after this period. Flow cytometry studies further confirmed the efficient intracellular uptake of Au‐R12P (Figure S9, Supporting Information). We then employed Raman confocal microscopy to assess the capabilities of Au‐R12P in distinguishing IDH genotypes by generating intracellular distribution maps of aiming metabolites (**Figure** **4**A). Notably, glioma cells exhibited significantly higher concentrations of intracellular GSH (IDH1‐WT GL261: 10.17 ± 1.45 mm, IDH1‐MUT GL261: 8.56 ± 0.67 mm) compared to normal brain cells (astrocytes: 1.78 ± 0.33 mm, neurons: 2.21 ± 1.05 mm, macrophages: 1.28 ± 0.40 mm) as measured by the I_542_/I_928_ ratio (Figure 4B,D; Figure S10, Supporting Information). To further distinguish IDH1 genotypes of glioma cells, we delineated intracellular H_2_O_2_ distribution maps by measuring the I_628_/I_928_ ratio pixel by pixel. Elevated H_2_O_2_ concentration (114 ± 19.94 µm) in IDH‐MUT GL261 cells was observed, surpassing those of IDH‐WT GL261 cells by 1.62 times (Figure 4C,E; Figure S11, Supporting Information). The H_2_O_2_/GSH ratio mapping of live glioma cells clearly differentiates their IDH1 genotype. Validation using GSH and H_2_O_2_ assay kits further confirmed the accuracy of Au‐R12P in detecting both metabolites (Figure 4D,E). To further validate the robustness of our method, we expanded our approach to include patient‐derived IDH‐mutant cell lines. Specifically, we used TS603 (an oligodendroglioma cell line with the IDH1‐R132H mutation) and HT1080 (a fibrosarcoma cell line with the IDH1‐R132C mutation). For comparison, we included U251 (a human glioma cell line with wild‐type IDH) and RD (a rhabdomyosarcoma cell line with wild‐type IDH) as controls. Raman confocal microscopic imaging showed that TS603 and HT1080 cells exhibited lower I_628_/I_928_ ratios, indicating elevated H₂O₂ levels compared to the wild‐type cells (U251 and RD) (Figure 4F,G; Figure S12, Supporting Information). The H₂O₂/GSH ratio further corroborated this difference, highlighting the elevated oxidative stress in the IDH‐mutant cells. These findings were also confirmed using a commercial H₂O₂ assay, which showed similar results, reinforcing the accuracy of our detection method in patient‐derived IDH‐mutant cell lines (Figure S13, Supporting Information). Collectively, these experiments underscore the ability of Au‐R12P to differentiate IDH1 genotypes of glioma cells at the cellular level.

To validate whether the detection method reflects the neomorphic activity of the IDH mutant enzyme, we treated tumor cell lines (TS603, HT‐1080, U251, and RD) with the IDH mutant enzyme inhibitor ivosidenib (650 nm) for 48 h to selectively inhibit the neomorphic activity of the IDH mutant enzyme. Raman confocal images show that the H₂O₂/GSH ratios in TS603 and HT‐1080 cells treated with ivosidenib are significantly lower compared to the untreated group. Conversely, IDH1‐WT RD and U251 cells exhibit similar results between the ivosidenib‐treated and PBS‐treated groups (Figure 4F–I, Figure S14, Supporting Information). This suggests that the inhibitory effect of ivosidenib on redox changes is evident in IDH1‐mutated TS603 and HT‐1080 cells, but not in IDH1 wild‐type RD and U251 cells. We also observed similar results using a commercial H₂O₂ detection assay (Figure S13, Supporting Information). The H₂O₂ levels in TS603 and HT‐1080 cells decreased significantly after ivosidenib treatment, confirming that the redox changes were predominantly driven by mutant IDH activity.

### 2.5 Au‐R12P Shows Specificity to Glioma Allografts Regardless of their IDH Genotypes

**Figure 5:**
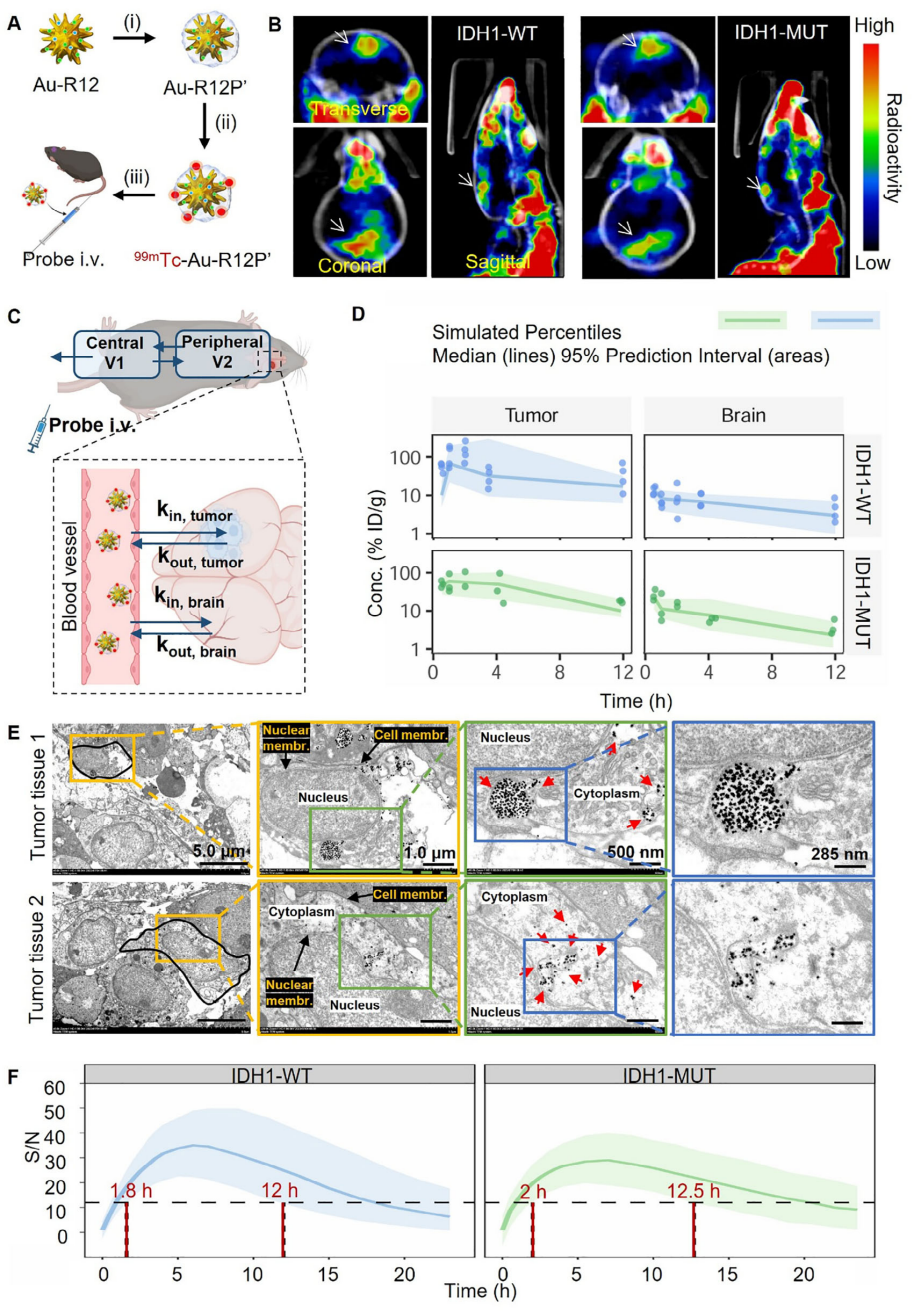
Au‐R12P visualizes IDH‐WT and IDH‐MUT glioma allografts after intravenous administration. A) SERS probe Au‐R12P was radio‐labelled with ^99m^Tc and then injected intravenously into mouse models bearing orthotopic IDH1‐WT/MUT glioma allograft. (i) HS‐PEG^5k^‐DTPA, 12 h, r.t.; (ii) ^99m^Tc^3+^, 1.0 h, 50 °C; (iii) 15000 rpm, 10 min, r.t. P' refers to the thiol‐DTPA derivative (SH‐PEG^5K^‐DTPA). Created with BioRender.com. B) Representative SPECT/CT images of IDH1‐WT/MUT glioma models after the intravenous administration of ^99m^Tc‐Au‐R12P' (200 µCi/mouse). Arrows point to the glioma position in the brain. C) PK model consists of a two‐compartment disposition of ^99m^Tc‐Au‐R12P' in blood, as well as the biodistribution of ^99m^Tc‐Au‐R12P' among blood, normal brain, and tumor allograft. Created with BioRender.com. D) Biodistribution of ^99m^Tc‐Au‐R12P' in IDH1‐WT/IDH1‐MUT tumor allograft and normal brains (*n* = 4/group) at selected time points post‐injection. E) TEM images of GL261 glioma tissues and normal brain tissues of mouse models at 8 h post intravenous injection of Au‐R12P (1.50 nmol g^−1^). The black curve in the leftmost panel depicts the outline of the cell. Black arrows indicate cell or nuclear membranes, and red arrows indicate probe Au‐R12P. Scale bars: 5.0 µm (leftmost panel), 1.0 µm (second‐left panel), 500 nm (second‐right panel), and 285 nm (rightmost panel). F) Time‐dependent signal‐to‐noise ratio (S/N) of Raman spectra after intravenous administration of Au‐R12P (1.50 nmol g^−1^) in mouse models bearing IDH1‐WT or IDH1‐MUT glioma allograft. The black dashed line indicates the ideal S/N value, meanwhile, the time slot between the two red lines is the recommended time window for in vivo Raman signal measurement.

Single‐photon emission computed tomography (SPECT) imaging was used to investigate the distribution and glioma‐targeting delivery of Au‐R12P in vivo. Au‐R12P labelled with radioisotope ^99m^Tc (^99m^Tc‐Au‐R12P') was synthesized and administered intravenously (i.v.) into mouse models bearing intracranial glioma IDH1‐WT and IDH1‐MUT allografts (**Figure** **5**A). SPECT/CT imaging revealed the preferential accumulation of ^99m^Tc‐Au‐R12P' in both IDH1‐WT and IDH1‐MUT GL261 allografts, but minimal presence in normal brain tissue (Figure 5B). We further investigated the pharmacokinetics (PK) and pharmacodynamics (PD) of ^99m^Tc‐Au‐R12P' in animal studies (Figure S15A, Supporting Information). The PK model consisted of a two‐compartment disposition of ^99m^Tc‐Au‐R12P' in the blood, as well as the biodistribution among blood, normal brain, and tumor allograft (Figure 5C). Compared to the normal brain tissue, a distribution of this probe into gliomas was evident regardless of IDH1 genotype (Figure 5D). Then we labeled Au‐R12P with CY5 fluorophores (Figure S16, Supporting Information), which were intravenously injected into tumor‐bearing mice. Fluorescence confocal imaging of brain sections revealed significant probe accumulation in the tumor region. This finding was consistent with the TEM images in murine brain tissues, where numerous probes with stellate structures were located in the IDH1‐WT/MUT glioma regions, in contrast to their minimal visibility in the normal brain regions (Figure S17, Supporting Information). Larger field‐of‐view TEM images reveal Au‐R12P was encapsulated within endocytic vesicles, with some probes escaping into the cytoplasm (Figure 5E). These observations suggested that the probe is internalized through endocytosis and primarily exerts its function within the cytoplasm. In addition, the biodistribution of the probes in major organs, including the heart, liver, spleen, lungs, kidneys, stomach, and intestine, showed consistency between IDH1‐WT and IDH1‐MUT models (Figure S15B, Supporting Information). Furthermore, we developed a PD model to study the signal‐to‐noise (S/N) ratio of the Raman signal over time after intravenous injection of Au‐R12P. The simulation from the final PK/PD model indicated that the optimal imaging window was between 2 to 12 h post‐injection of Au‐R12P (1.50 nmol g^−1^) (Figure 5F).

### 2.6 Au‐R12P Identifies IDH1 Genotype of Glioma Allograft/Xenograft in Animal Models

**Figure 6:**
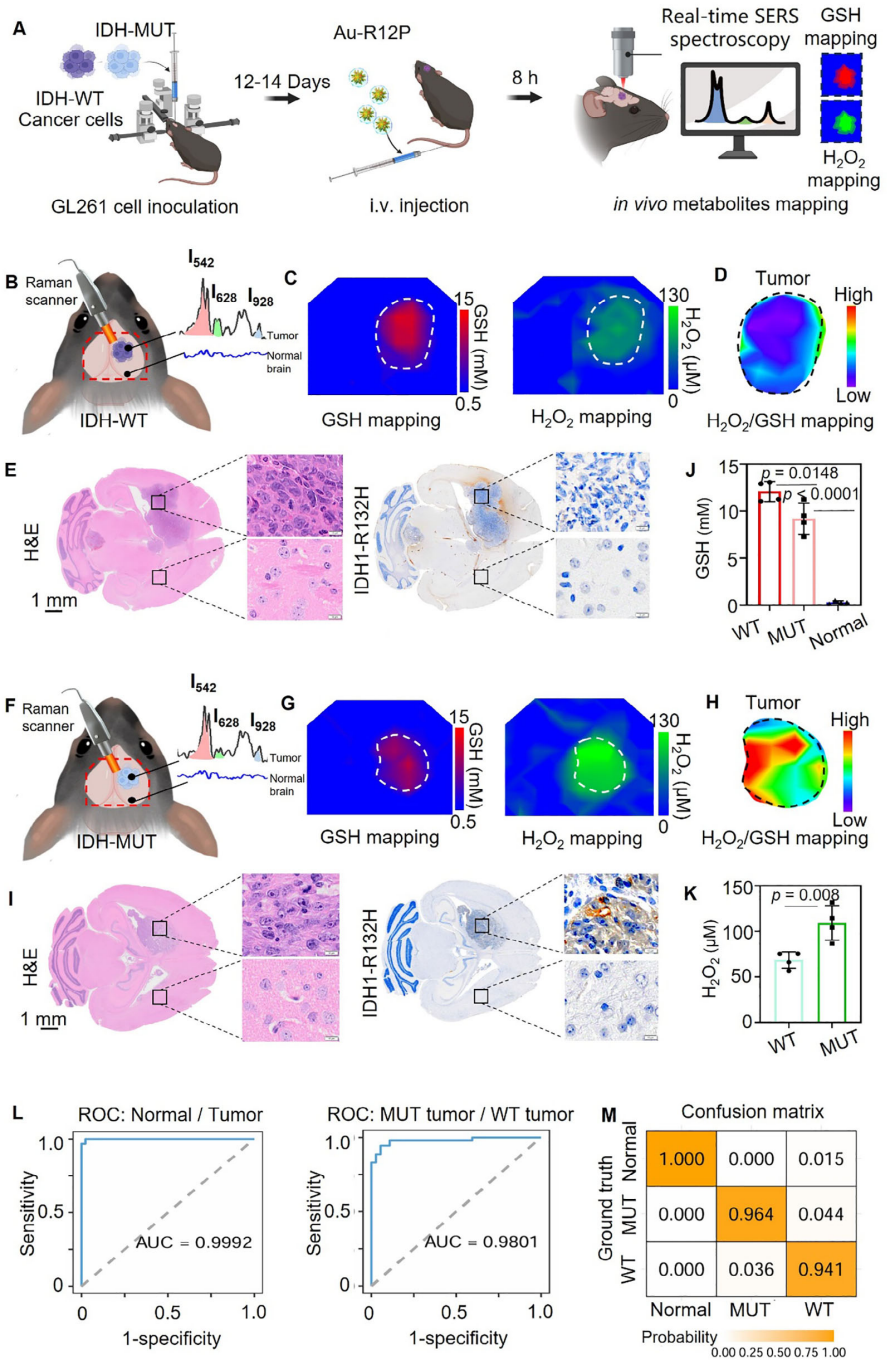
Au‐R12P classifies IDH genotypes of glioma allografts in animal models. A) Schematic diagram of in vivo SERS imaging workflow. Au‐R12P was injected in mouse models bearing IDH1‐WT or IDH1‐MUT glioma allograft. Raman signal on the brain region was detected by a handheld Raman detector at 8 h post administration of the probe. Created with BioRender.com. B) Diagram of intraoperative Raman imaging of GL261 glioma allograft during craniotomy. Insert: Raman spectra of IDH1‐WT tumor and surrounding normal brain tissue post administration of Au‐R12P. C) In vivo GSH and H_2_O_2_ distribution maps of the exposed tumor region in IDH1‐WT glioma models. D) The distribution map of H_2_O_2_/GSH ratio in IDH1‐WT tumor region at 8 h post probe administration. E) Histopathologic H&E and IDH1‐R132H immune‐staining images of whole brain sections from mouse models bearing IDH1‐WT glioma. F) Diagram of intraoperative Raman imaging of GL261 glioma allograft during craniotomy. Insert: Raman spectra of IDH1‐MUT tumor and surrounding normal brain tissue. G) In vivo GSH and H_2_O_2_ distribution maps of the exposed tumor region in IDH1 mutant glioma models. H) The distribution map of H_2_O_2_/GSH ratio in IDH1‐MUT tumor region at 8 h post probe administration. I) Histopathologic H&E and IDH1‐R132H immune‐staining images of whole brain sections from mouse models bearing IDH1 mutant glioma. Average GSH J) and H_2_O_2_ K) concentrations in tumor and contralateral normal brain tissue (*n* = 4). L) The results from the logistic regression model with I_542_/I_928_ ratios and I_628_/I_928_ ratios as explainable variables for the classification of normal brain and glioma with different IDH1 genotypes. The ROC curve illustrates the performance of Au‐R12P in discriminating tumor tissue (left) and IDH1 genotypes (right) in vivo. The AUC are 0.9992 and 0.9801, respectively. M) Confusion matrix of the three diagnostic subtypes. Normal: normal brain; WT: IDH1‐wild type glioma; MUT: IDH1‐mutant glioma. Data are shown as mean ± S.D. The Ordinary one‐way ANOVA is used for the multiple comparisons of three sets of data in J. An unpaired t‐test is used to compare two sets of data in K. Statistical significance is determined for a *p*‐value of <0.05.

Subsequently, we tested the capability of Au‐R12P in intraoperatively discerning IDH1 genotypes by using a handheld Raman scanner. Point‐by‐point scanning was performed on an area of roughly 0.8 × 0.8 cm in the horizontal plane of the mouse brain (including the tumor region and surrounding normal brain tissue), and 15‒20 scanning points were completed in 2 min (**Figure** **6**A). Compared to the weak Raman signals observed in normal brain, the signals from Au‐R12P were pronounced in tumor regions (Figure 6B,F). In vivo distribution maps of GSH or H_2_O_2_ were generated by collecting the ratios of I_542_/I_928_ or I_628_/I_928_, respectively (Figure 6C,G). The average GSH concentration was determined as 12.08 ± 0.53 mm in IDH1‐WT and 9.20 ± 0.83 mm in IDH1‐MUT GL261 allografts (Figure 6J). In contrast, the average GSH concentration in normal brain was below 1.0 mm. Furthermore, the average H_2_O_2_ concentration was measured at 68.54 ± 4.51 µm in IDH1‐WT GL261 tumor and 109.3 ± 9.44 µm in IDH1‐MUT GL261 tumors (Figure 6K). The accuracy of Au‐R12P in detecting both metabolites was further validated through GSH and H₂O₂ assay kits (Figure S18, Supporting Information). Given the elevated H_2_O_2_ and reduced GSH levels in IDH1‐MUT GL261 tumor, H_2_O_2_/GSH mapping serves as a parameter to differentiate glioma genotypes (Figure 6D,H). Hematoxylin and eosin (H&E) staining affirmed the feasibility of Au‐R12P to distinguish tumor regions from normal brains (Figure 6E,I). Furthermore, immunohistochemistry staining with the IDH1 mutant enzyme antibody IDH1‐R132H indicated that Au‐R12P effectively differentiates between IDH1‐WT and IDH1‐MUT gliomas allografts. From receiver‐operating characteristics (ROC) curves, the resulting area under the curve (AUC) values for distinguishing tumor from normal brain tissue and for differentiating the glioma IDH1 genotypes were 0.9992 and 0.9801, respectively (Figure 6L). In addition, the confusion matrix (Figure 6M) confirmed that Au‐R12P has great diagnostic performance in determining IDH1 genotypes (Predictive accuracy: 96.4% for IDH1‐MUT glioma; 94.1% for IDH1‐WT glioma).

Next, we validated our method in orthotopic xenograft nude mouse models using TS603 and U251 cells (Figure S19A, Supporting Information). The in vivo distribution of H₂O₂ and GSH was visualized by collecting the I_542_/I_928_ and I_628_/I_928_ ratios (Figure S19B–D,F–H, Supporting Information). We found that the average GSH concentration was 12.77 ± 0.56 mm in U251 (IDH1‐WT) tumors and 9.38 ± 0.87 mm in TS603 (IDH1‐MUT) tumors, whereas the GSH levels in normal brain tissue were below 1.0 mm (Figure S19J, Supporting Information). Additionally, the average H₂O₂ concentration was measured at 41.88 ± 10.92 µm in U251 (IDH1‐WT) tumors and 87.83 ± 3.41 µm in TS603 (IDH1‐MUT) tumors (Figure S19K, Supporting Information). These findings indicate a marked increase in oxidative stress in the IDH‐mutant tumors. The H₂O₂/GSH mapping successfully distinguished glioma genotypes, with an ROC curve showing an AUC of 0.9094 for differentiating the genotypes (Figure S19L, Supporting Information). Furthermore, H&E staining confirmed the feasibility of using the Au‐R12P probe to distinguish tumor regions from normal brain tissue (Figure S19E,I, Supporting Information). Overall, Au‐R12P demonstrates potential in intraoperatively identifying IDH1 genotypes of glioma by measuring redox metabolites.

Then, we conducted in vivo experiment where ivosidenib (50 mg kg^−1^), an IDH1 mutant enzyme inhibitor, was orally administered to TS603 (IDH1‐MUT) and U251 (IDH1‐WT) tumor‐bearing nude mice (Figure S20A, Supporting Information). Raman signal measurements were taken from the brain regions 12 hours post‐treatment to assess the impact of the inhibitor on oxidative stress (Figure S20B–E,F–I, Supporting Information). The results showed that after inhibition of the IDH1 mutant enzyme with ivosidenib, the average H₂O₂ concentration was measured at 51.25 ± 8.94 µm in IDH1‐WT (U251 + ivosidenib) tumors and 46.96 ± 9.15 µm in IDH1‐MUT (TS603 + ivosidenib) tumors (Figure S20J, Supporting Information), with no significant difference between the two groups. Similarly, the H₂O₂/GSH profiles showed no significant difference between the treated IDH1 wild‐type and mutant tumors (Figure S20L, Supporting Information). Moreover, H₂O₂ levels measured using a commercial assay in the tumor tissues corroborated these results (Figure S20K, Supporting Information). These findings indicate that inhibition of the IDH1 mutant enzyme reduces the oxidative stress levels in IDH1‐mutant gliomas.

### 2.7 Au‐R12P Intraoperatively Identifies IDH1 Genotype of Patient Glioma

**Figure 7:**
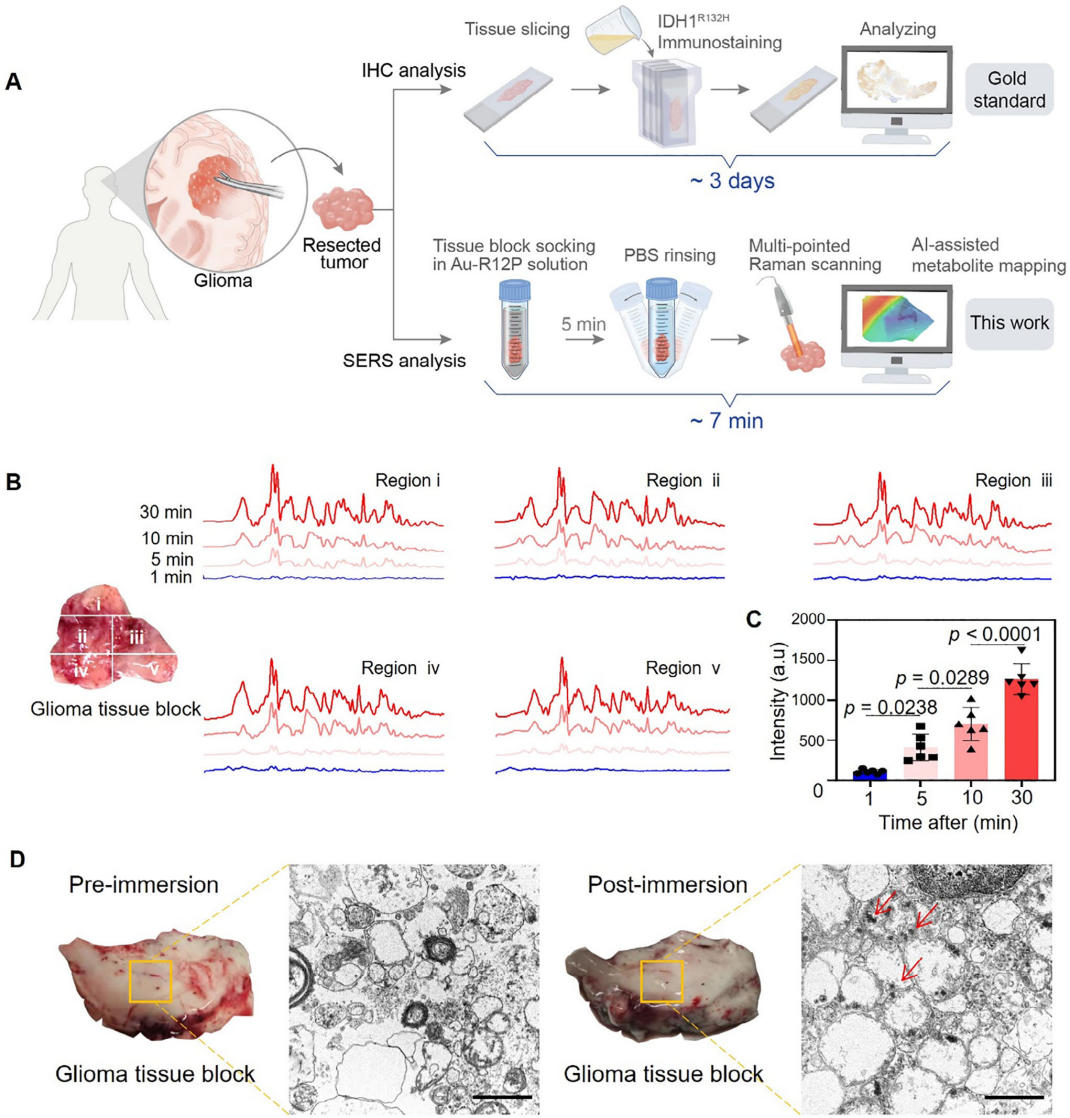
Au‐R12P penetrates into excised glioma tissue from patients after soaking treatment. A) The experimental workflow for intraoperatively identifying IDH1 genotypes of glioma patients. The excised glioma tissue block was immersed in a saline solution of Au‐R12P. The tissue was rinsed by saline to remove the non‐penetrated probes. The Raman signals were detected using a handheld Raman instrument to generate GSH and H_2_O_2_ distribution images. B) Representative Raman spectra of tissue immersed in Au‐R12P solution for 1.0, 5.0, 10, and 30 min. The Raman spectra were collected from 5 regions of the excised tissue block. C) Average Raman intensities collected from the glioma tissue at selected times immersed in Au‐R12P solution. *n* = 6. D) TEM images of human glioma tissue pre‐ and post‐immersion of Au‐R12P solution for 5 min. Scale bar: 1.0 µm.

The capability of Au‐R12P in distinguishing IDH1 genotypes was evaluated in the operating room settings. After collecting the freshly excised tumor tissue block, we immediately immersed it in a saline solution of Au‐R12P (20 µm) followed by rinsing it with saline three times (**Figure** **7**A; Figure S21, Supporting Information). The Raman signals of the tissue block were measured multi‐pointedly using a handheld Raman scanner. We first investigated the immersion duration required to generate a detectable SERS signal on the tumor tissue surface. As shown in Figure 7B,C, an obvious Raman signal on the patient tissues was observed as soon as 5 min after immersion, with the signal intensity increasing over time and peaking at 30 min. While the Raman signal intensities of I_542_, I_628_, and I_928_ increased with soaking time, the calculated ratios (I_542_/I_928_ for GSH and I_628_/I_928_ for H₂O₂) remained stable, especially after 4 min (Figure S22, Supporting Information). This indicates that the ratiometric method effectively corrects handling‐induced variability and provides reliable detection of both metabolites. TEM images confirmed the intracellular uptake of Au‐R12P on the tissue surface (Figure 7D). After balancing the trade‐off between immersion time and signal reliability, we chose an immersion time of 5 min for Raman signal measurement. To verify whether tissue immersion method produces yields results consistent with the probe i.v. administration method used in animal experiments, we compared the H₂O₂ results obtained using both methods (Figure S23, Supporting Information). The similar H₂O₂ levels suggested that the sample handling process (soaking vs injection) does not introduce significant variability.

**Figure 8:**
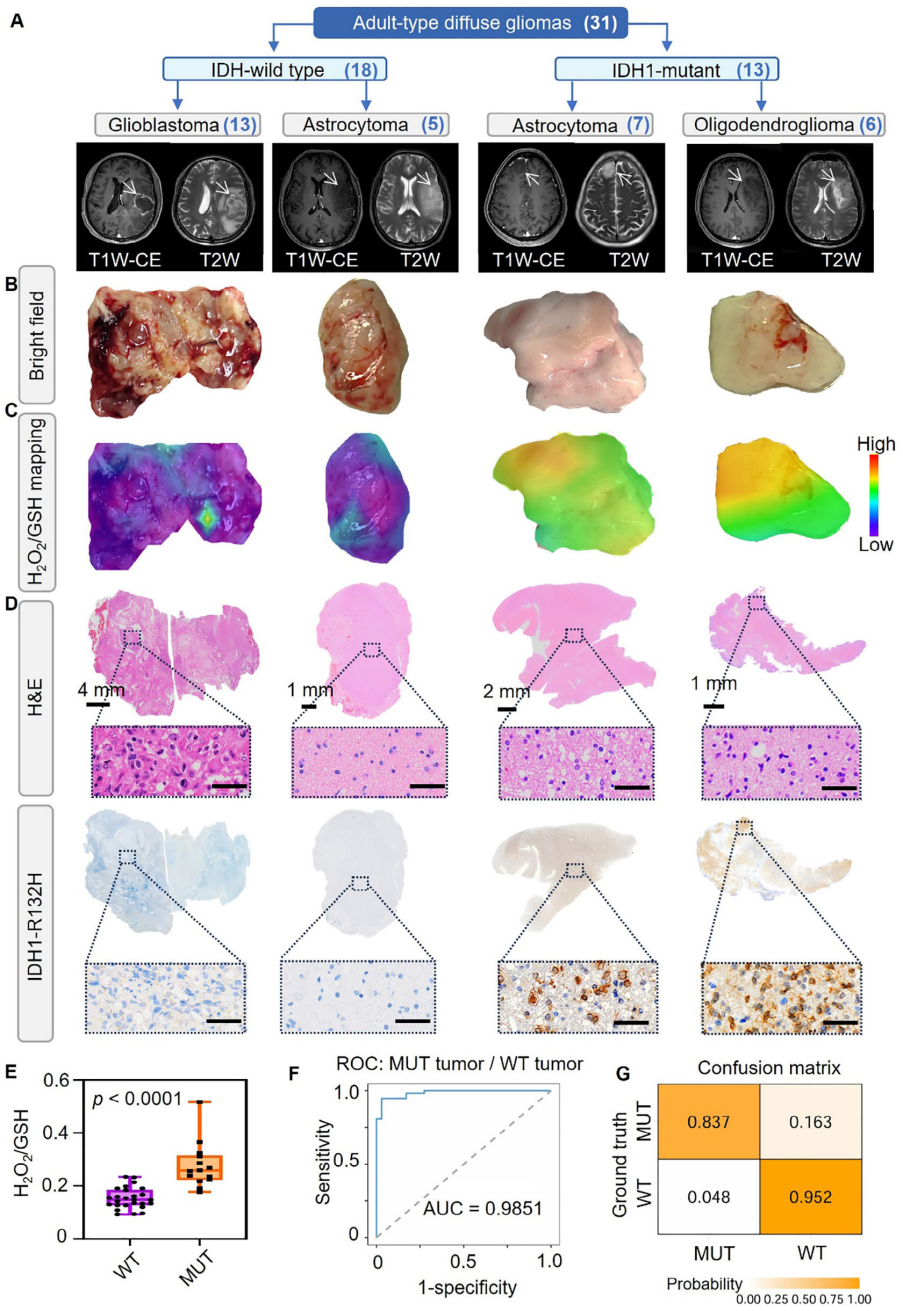
Au‐R12P intraoperatively classifies IDH1 genotypes of glioma from patients. A) According to the fifth edition of the World Health Organization's (WHO) classification of tumors of the central nervous system (CNS), adult‐type diffuse gliomas are divided into: Glioblastomas, IDH‐wild type; Astrocytomas, IDH‐wild type; Astrocytomas, IDH mutant; Oligodendrogliomas, IDH‐mutant and 1p/19q co‐deletion. Representative T1W‐CE and T2 W MRI images of the corresponding glioma classification are shown below. B) Representative bright field photographs of the freshly excised patients’ glioma tissues belonging to the above glioma categories. C) Representative H_2_O_2_/GSH ratio distribution maps of the excised glioma tissues. GSH and H_2_O_2_ distribution images were generated by calculating the Raman peak ratios of I_542_/I_928_ or I_628_/I_928_ at the multiple sampling points on the excised glioma tissues. D) Histological H&E and IDH1‐R132H immunohistochemical staining of the isolated glioma tissues demonstrated in panel b (amplified image. Scale bar: 40 µm). E) Comparison of average H_2_O_2_/GSH ratios in IDH1‐WT and IDH1‐MUT glioma tissues. (WT: 26 tissues from 18 patients. MUT: 14 tissues from 13 patients.) F) The ROC curve of the logistic regression model with H_2_O_2_/GSH as explainable variables for the classification of IDH1‐WT/MUT gliomas in clinical setting. G) Confusion matrix of the two glioma subtypes. WT: IDH1‐wild type glioma; MUT: IDH1‐mutant glioma. Data are shown as mean ± S.D. An unpaired t‐test is used to compare two sets of data in E. Statistical significance is determined for a *p*‐value of <0.05.

According to the fifth edition of the WHO classification of Tumors of the Central Nervous System (CNS), we collected a cohort comprising 31 patients diagnosed with adult‐type diffuse gliomas. This cohort comprised 18 cases of IDH1‐WT gliomas (13 glioblastoma, 5 astrocytoma) and 13 cases of IDH1‐MUT gliomas (7 astrocytoma, 6 oligodendrogliomas) (**Figure** **8**A, Table S3, Supporting Information). Figure 8B displays the bright‐field images of resected tissue blocks from glioma patients with different subtypes, along with the intraoperatively generated H_2_O_2_/GSH ratio distribution maps (Figure 8C). Regardless of IDH1 genotype, glioma tissues exhibited much higher levels of GSH compared to adjacent non‐malignant tissues. Notably, IDH1‐MUT glioma tissues exhibited higher concentrations of H_2_O_2_ compared to IDH1‐WT tissues (Figure S24, Supporting Information). Meanwhile, this difference was more pronounced in the H_2_O_2_/GSH profiles, which was 1.81‐fold higher than that of IDH1‐WT (Figure 8E). Subsequently, both H&E staining and IDH1‐R132H immunohistochemistry results verified the capability of Au‐R12P in identifying IDH1 genotypes of glioma tissues (Figure 8D). Furthermore, the ROC curve of the H_2_O_2_/GSH incorporated logistic regression model indicated the performance of Au‐R12P in classifying glioma IDH1 genotypes in clinical settings, with an AUC of 0.9851 (Figure 8F). As shown in the confusion matrix, Au‐R12P demonstrated a predictive accuracy of 0.837 for IDH1‐MUT and 0.952 for IDH1‐WT (Figure 8G). The studies above demonstrated the feasibility of using Au‐R12P for differentiating IDH1 phenotypes in clinically relevant scenarios.

## 3 Discussion

IDH mutations are recognized in more than 70% of WHO low‐grade gliomas, typically diagnosed in adults younger than 50‐year‐old. While less common in primary WHO high‐grade glioblastoma multiforme (GBM) (3.7%), these mutations frequently appear in recurrent GBM, accounting for 73% of cases. The treatment for LGGs involves maximally safe tumor resection, followed by radiation and chemotherapy as indicated. Although IDH‐mutated gliomas have a better prognosis compared to their IDH wild‐type counterparts, they are not curative, and most patients will eventually experience recurrence and progression to a higher grade. Recently, a phase 3 clinical trial showed that vorasidenib, greatly extended progression‐free survival and delayed the time for the next intervention.^[^ ^25^ ^]^ Therefore, intraoperatively identifying IDH mutation state is crucial, as it not only aids in timely formulating the surgical strategies, but also accelerates the clinical translation of IDH mutation inhibitors by providing patient stratification.

Developing SERS probes for simultaneous detection of multiple metabolites in biological systems is challenging, as the Raman reporters must meet the following criteria: 1) high responsiveness to the target metabolites; 2) orthogonal‐specificity to multiple metabolites; and 3) accurate measurement of metabolite concentrations. Tian's group^[^ ^18^ ^]^ developed a SERS optophysiological probe for the simultaneous mapping of the carbonate and pH in a live mouse brain. However, the applied Raman reporters, including 1‐(4‐aminophenyl)‐2,2,2‐trifluoroethanone and 4‐mercaptobenzoic acid, had small Raman scattering cross‐sections, resulting in limited sensitivity. We previously reported pH‐responsive SERS probes with detection limits as low as 0.8 pM using heptamethine cyanine derivative as the Raman reporter,^[^ ^26^ ^]^ but these were limited to single‐metabolite detection. In this study, we designed a ratiometric SERS probe to orthogonally determine the intracellular concentrations of GSH and H_2_O_2_ with the following features. First, heptamethine cyanine and rhodamine, with large Raman cross‐sections but different molecular skeletons, were selected as Raman reporters. The absorbance of these reporters matches the excitation laser wavelength (785 nm) and the energy to an electron transition of the gold nanoparticles, triggering the SERRS effect that further amplifies the sensitivity. Second, the employed reporter molecules RR1 and RR2 orthogonally respond to GSH and H_2_O_2_, respectively, with minimal interference from the circumstance of co‐existing these two metabolites. Third, the Raman peak of RR1 and RR2 show minimized interferences from the physiological ions and biological species, ensuring reliable determination of the corresponding metabolites. In addition, the intensity of the Raman peak at 928 cm^−1^ remains constant regardless of the metabolite concentrations, serving as an internal reference for ratiometric normalization and simplifying probe design by eliminating the need for a separate reference molecular reporter. Notably, our probe is designed to assess IDH1 mutations in excised tumor tissues rather than to locate tumor infiltration or deep‐seated tumors during surgery.

Machine learning algorithms are widely utilized for the quantitative or qualitative analysis of Raman spectroscopy, employing methods such as linear regression,^[^ ^27^ ^]^ support vector regression,^[^ ^28^ ^]^ K‐nearest neighbor,^[^ ^29^ ^]^ and linear discriminant analysis.^[^ ^30^ ^]^ Many studies have applied the convolutional neural networks (CNNs) commonly used in computer vision to the field of Raman spectroscopy. Most of them designed customized 1D‐CNNs, and a few studies converted the spectra into images, using 2D‐CNNs for spectral analysis.^[^ ^22^ ^]^ However, most existing regression models have been applied to the quantification of a single analyte. In this work, we constructed a 2D neural network, DBCNet, to simultaneously detect the concentration of two metabolites, GSH and H_2_O_2_. Compared with the current models, our method offers several advantages: 1) We transform the spectrum into images by RPM, which improves the richness of the feature representation and improves the model's efficacy in feature extraction. 2) We design a dual branch structure to decouple the training processes for the concentration detection of two metabolites, promoting specialized feature learning for each. 3) We propose a batch correlation module that enables mutual interaction of spectral features within the same batch, further improving the separability of different types of samples. The DBCNet model has broad applicability and does not require retraining for each individual clinical case.

Taking advantage of the multiplexity of Raman spectroscopy and the multi‐point sample collection during the surgery, we generated metabolite distribution maps on the surface of tissue blocks. Importantly, we showed the spatial heterogeneity of IDH1 mutation in human glioma tissue. This phenomenon can be explained by the differential cancer cell densities in the tissue. The region with a high density of IDH mutant cancer cells leads to a high H_2_O_2_/GSH ratio. Additionally, IDH mutations often co‐occur with glioma‐associated genetic alterations including BRAF, TERT promoter, 1p/19q co‐deletion, and MGMT promoter methylation,^[^ ^31^ ^]^ which may also result in shifts of metabolic pathways and the spatial heterogeneity of H_2_O_2_/GSH ratio. One study demonstrated that IDH mutations, when occurring alongside alterations like 1p/19q co‐deletion, profoundly impact glioma metabolism and tumor behavior.^[^ ^32^ ^]^ Even though the clinical relevance of visualizing the spatial heterogeneity of IDH1 mutation remains unclear, we anticipate that it provides a valuable parameter in intraoperatively evaluating tissue malignancy and invasiveness.

## 4 Conclusion

In this work, we present an AI‐assisted strategy to identify IDH1 genotypes of glioma by determining the redox‐associated metabolites. With assistance of the ratiometric SERS nanoprobe and the deep learning algorithm, we can intraoperatively identify IDH1 mutation status of glioma patients by soaking freshly excised glioma tissue in the aqueous solution of the nanoprobe, followed by point‐by‐point Raman signal acquisition on the tissue block surface. By analyzing 643 sample points from 31 glioma patients, we rapidly identify IDH1 genotypes with an area under the ROC curve of 0.985. This strategy enables the operator timely deduce the molecular subtype and pathological classification of glioma, thereby optimizing the surgical strategy and post‐operative treatment portfolio.

## 5 Experimental Section

### Patients and Tissue Specimens

This research protocol was approved by the Ethics Committee of Huashan Hospital of Fudan University (No.603, 2023) and is in accordance with the ethical guidelines of the Declaration of Helsinki (1975). The Ethical Approval Consent number was KY2023‐603. All patients signed informed consent prior to the study. The inclusion criteria for human specimens were patients scheduled for glioma resection. The exclusion criteria were patients who did not wish to undergo this test.

### Synthesis of the SERS Probe Au‐R12P

The Au‐ST was prepared as previously reported.^[^ ^33^ ^]^ Briefly, 100 mL of boiling aqueous gold chloride (HAuCl_4_, 1.0 mm) solution was quickly added with 15 mL 1% (w/v) sodium citrate in order to create 12 nm gold seeds. HAuCl_4_ (0.27 mm), HCl (0.12 mm), and gold seed solution (4.2 mL) were added to 200 mL of aqueous solutions stirred at 650 rpm. AgNO_3_ (3.0 mm) and L‐Ascorbic acid (100 mm) were then added to the mixture simultaneously with continuous stirring (1300 rpm) for 40 s to yield gold nanostars (Au‐ST). The Au‐ST solution was gently stirred for 1 h after the addition of RR1 and RR2 in a concentration ratio of 2:3. After the addition of HS‐PEG^5k^‐OCH_3_ and the purification in a 10 000 MW cutoff dialysis bag, the desired SERS probe was obtained.

### Synthesis of the SERS Probe Au‐RR1, Au‐RR2, and Au‐R12

The Au‐ST was prepared as previously reported. Briefly, 100 mL of boiling aqueous gold chloride (HAuCl_4_, 1.0 mm) solution was quickly added with 15 mL 1% (w/v) sodium citrate in order to create 12 nm gold seeds. HAuCl_4_ (0.27 mm), HCl (0.12 mm), and gold seed solution (4.2 mL) were added to 200 mL of aqueous solutions stirred at 650 rpm. AgNO_3_ (3.0 mm) and L‐Ascorbic acid (100 mm) were then added to the mixture simultaneously with continuous stirring (1300 rpm) for 40 s to yield gold nanostars (Au‐ST). Add RR1 (40 µL, 4 mm), RR2 (40 µL, 6 mm), RR1 (40 µL, 4 mm) +RR2 (40 µL, 6 mm) to the Au‐ST solution, respectively, and stir for one hour at room temperature. After ultracentrifugation at 11 000 rpm for 15 min, the supernatant was discarded to obtain the SERS probes Au‐RR1, Au‐RR2, and Au‐R12, respectively.

### Raman Spectra of the SERS Probe Au‐RR1, Au‐RR2, and Au‐R12

The Raman spectra of Au‐RR1, Au‐RR2, and Au‐R12 were acquired by A portable hand‐held Raman spectrometer (QE65, Ocean Optics, Florida) with a 785 nm excitation laser (laser power: 10 mW, grating: 600 gr mm^−1^, acquisition time: 500 ms). The acquired Raman spectra were disposed of using OceanView software (Version 1.6.7) and LabSpec5 software (Version 5.58.25).

### Cell Lines

Mouse glioblastoma (GBM) cell line GL261 glioma cells, C8‐D1A astrocyte, HT22 neuron, and RAW 264.7 macrophage cells were obtained from American type culture collection. The IDH1‐WT and IDH1‐MUT GL261 cells used in this study were derived from previous work conducted by the research group.^[^ ^34^ ^]^ Human glioma U251 (IDH1‐WT) cell line was generously provided by Professor Chen Liang from the Department of Neurosurgery at Huashan Hospital. Human oligodendroglioma TS603 (IDH1‐MUT) cell line was purchased from Qingqi (Shanghai) Biotechnology Development Co. Human fibrosarcoma HT1080 (IDH1‐MUT) cell line was purchased from Servicebio (Wuhan) Biotechnology Co. Human rhabdomyosarcoma RD (IDH1‐WT) cell line was generously provided by Professor Jie Xu from school of pharmacy, Fudan university, China.

### Dataset and Data Preprocessing

The Raman spectral database contains 9600 spectra totally. Specifically, the concentration values of GSH were selected as 2, 4, 6, 8, 10, and 16 mm, while the concentration values of H_2_O_2_ were selected as 1, 25, 50, 75, 100, 125, 150, and 200 µm. Two hundred SERS spectra of standard buffer solution were collected for each H_2_O_2_ concentration under different GSH concentrations. The 50^th^ to 336^th^ points of the original Raman spectra were kept to reduce computational complexity. Then, each spectrum was divided by its maximum value as normalization.

### DBCNet Model Architecture and Training Details

The preprocessed spectra were first converted into the RPM as Equation (3).

(3) $$RPM_{i,j}=x_{i}\;-x_{j},i,j=1,2,\text{…},N$$

where *N* is the length of the spectrum, and *x_i_* is the *ith* point of the spectrum.

Next, the 2D Raman images were fed into the ResNet‐18 backbone for feature extraction. The output of the last global average pooling layer in ResNet‐18 was employed as the shared feature vector of GSH and H_2_O_2_ for the subsequent network. Then, the refined features of GSH and H_2_O_2_ were learned separately by batch correlation. Specifically, the feature similarity between pairwise spectra was measured in a batch and update the features for GSH (or H_2_O_2_) as Equation (4).

(4)

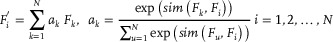

where *N* is the size of batch, *F_k_* is the original *kth* feature in the batch, $F_{i}^{\prime }$ is the updated *ith* feature in the batch, *a_k_* is the weight that *F_k_* contributes to *F_i_*, *sim*(·) is cosine similarity.

After the batch correlation layer, the fully connected layer was employed to obtain the predicted concentration results of GSH and H_2_O_2_. The Nadam optimizer was used with the initial learning rate of 0.001, the MAE loss function, epoch of 400 and batch size of 32. The patience and factor of the learning rate reduction was 25 and 0.1, respectively, and the patience of early stopping was 30.

### Raman Confocal Studies

For cell samples, Raman spectra were acquired using a confocal Raman microscope (WITec Raman alpha300 R, WITec, Germany) in the standard mode at a temperature of ‐60 °C. The Raman laser excitation source employed was a diode laser operating at a wavelength of 785 nm. Raman spectra were captured using the following parameters: a 300 mm spectrograph equipped with a 300 g mm^−1^ grating, an excitation power of 17 mW, and a 63× objective (0.9 NA, Zeiss). Subsequently, the obtained data were subjected to analysis using both the WITec Project software (Version 5.2) and LabSpec5 software (Version 5.58.25).

### Animals and Tumor Models

C57/BL6 mice (males, 5–6 weeks old, 18–22 g) and BALB/c Nude mice (males, 6–8 weeks old) were obtained from Shanghai SLAC Laboratory Animals and kept under specific pathogen‐free (SPF) conditions at the Animal Experiment Center, School of Pharmacy, Fudan University. All animal experiments were conducted in strict accordance with the guidelines approved by the Ethics Committee of Fudan University (2022‐03‐FY‐LC‐45). Briefly, the mice were anesthetized using 2% isoflurane and secured in a stereotaxic apparatus (RWD, Shenzhen, China). IDH1‐WT/MUT GL261 cells (1.0 × 10^5^ cells in 5 µL PBS) or TS603/U251 cells (1.0 × 10^6^ cells in 5 µL PBS) were injected stereotactically into the right striatum of the mice using a 5 µL Hamilton syringe, with the following stereotaxic coordinates: 2.0 mm lateral, 1.0 mm anterior to bregma, and 3.2 mm deep. The syringe was carefully withdrawn from the brain, pausing at a depth of 2.0 mm for 1 min. These orthotopic glioma models were validated using T2W‐MR and were ready for experimentation once the tumor diameter exceeded 2 mm.

### Radio‐Synthesis of ^99m^Tc‐Au‐R12P’

Briefly, a mixture of Aminated polyethylene glycol derivative (NH_2_‐PEG^5k^‐NH_2_) (100 mg, 20 mmol), lipoic acid (4.12 mg, 20 mmol), and HATU (11.4 mg, 30 mmol) in 10.0 mL anhydrous DMF was stirred thoroughly with TEA (5 mg, 50 mmol) at r.t. for 48 h. Diethylenetriamine‐pentaacetic acid dianhydride (4.4 mg, 12.3 mmol) and TEA (5 mg, 50 mmol) were then added, followed by stirring at 40 °C for 48 h. The resulting reaction mixture was used directly in subsequent reactions without any further treatment. DTPA‐modified Au‐R12P’ was prepared in a manner similar to that of Au‐R12P, with the incorporation of a reaction mixture containing the thiol‐DTPA derivative. For radiolabeling with ^99m^Tc, DTPA‐modified Au‐R12P’ and stannous chloride (SnCl_2_, 50 µg, pH 2.0) were added to 1 mL of PBS (pH 6.5) and vortexed. Freshly eluted 99mTechnetium pertechnetate (0.2–0.5 mL, ≈200 MBq) was then introduced into the mixture and incubated at 50 °C for at least 30 min. The mixture was purified by centrifugation at 12 000 rpm for 10 min, repeated three times to eliminate any unlabeled ^99m^Tc.

### In Vivo SPECT/CT Studies

IDH1‐WT and IDH1‐MUT glioma mouse models underwent SPECT/CT imaging following intravenous administration of ^99m^Tc‐Au‐R12P (6.0−8.0 MBq) at 0.5, 2, and 6 h post‐injection. During the scanning period, mice were anesthetized with 1−2% isoflurane. Imaging was conducted with a nanoScan@SC SPECT/CT system (Mediso Medical Imaging System, Hungary), which features four high‐resolution conical collimators fitted with multi‐pinhole plates. For the CT image acquisition, parameters included a 0.98 mA 50 kVp x‐ray tube, 300 ms exposure time, and a total of 720 projections. SPECT imaging was conducted under standard mode with parameters including a 140 keV energy peak, 20% full width, 1 mm/pixel resolution, 256 × 256 matrix size, 30 s/projection scan time, and a total of 128 projections. Subsequently, all collected data were processed and evaluated using specialized fusion software (Version 3.00.021.000).

### In Vivo SERS Imaging in Mouse Models

The IDH1‐WT or IDH1‐MUT glioma mouse models were established following the methods detailed above. Ten days post‐inoculation of cancer cells, tumor volume was monitored using T2‐weighted magnetic resonance imaging (T2W‐MRI). Twelve to fourteen days after tumor cell implantation, the mice were injected intravenously via tail vein with Au‐R12P (1.50 nmol g^−1^). After an 8 h interval, the mice were anesthetized using 1–2% isoflurane and subjected to a craniotomy procedure to expose the cerebral cortex for subsequent Raman imaging. Spot scanning was performed in an area of ≈0.8 × 0.8 cm in the horizontal plane of the mouse brain (including normal and tumor areas), and the entire scanning process was completed in 2 min. Quantitative analysis of the acquired Raman spectra enabled the determination of the average GSH or H_2_O_2_ levels. This was achieved by calculating the intensity ratio of I_542_/I_928_ or I_628_/I_928_ and applying these values to the corresponding fitting formula. Guided by the generated GSH map in real‐time, tumor and non‐tumor regions were distinguished. And by the generated H_2_O_2_ map in real‐time, it was possible to determine whether the tumor regions were wild‐type or mutant. The spatial resolution of Raman images in animal experiments was 1.8–2.0 mm. The Raman spectra were acquired by A portable hand‐held Raman spectrometer (QE65, Ocean Optics, Florida) with a 785 nm excitation laser (laser power: 10 mW, grating: 600 gr mm^−1^, acquisition time: 500 ms, laser spot was 200 µm). The acquired Raman spectra were disposed using OceanView software (Version 1.6.7) and LabSpec5 software (Version 5.58.25).

### SERS Imaging of Resected Tumor Tissue from Glioma Patients

The size of the tumor tissue selected for experiments was determined by the size of the patient's tumor during surgery, with the maximum surface area of the tissue typically ranging from 0.25 to 9 cm^2^. Fresh glioma tissue could be detected within 7 min post‐resection. The procedure involved soaking freshly excised tissue samples in Au‐R12P in a PBS solution for 5 min, followed by three washes in PBS. Detection was carried out using a handheld Raman detector, with sampling points spaced ≈2.0–4.0 mm apart. The number of sampling points was determined based on the size of the tissue area to be tested, with ≈16 detection points on a 2 × 2 cm tissue sample. Raman spectra were collected using a portable Raman spectrometer (QE65, Ocean Optics, Florida) with a 785 nm excitation laser (laser power: 10 mW, grating: 600 gr mm^−1^, acquisition time: 500 ms, laser spot was 200 µm). The values for GSH, H_2_O_2_, and H_2_O_2_/GSH at each detection point were obtained through an automated program. Finally, the patient's tissues underwent immunohistochemical analysis post non‐destructive testing.

### Immunohistochemistry Studies

After rapid detection with a dual‐response SERS system, glioma tissues removed from patients were immersed in 4% paraformaldehyde (PFA). After being treated sequentially with dimethylbenzene, different concentrations of ethanol and water, paraffin Sections (5 µm) were made starting from the tissue detection surface. Then hematoxylin and eosin (H&E) staining and IDH1‐R132H (ZSGB‐Bio, ZM‐0447) staining were performed.

### Statistical Analysis

Statistical analysis was performed using Origin 2019b software (Microcal Software Inc., Northampton, USA), MATLAB (R2020b) and GraphPad Prism 8.0.2. All of the data were expressed as means ± standard deviation (S.D.). Statistical data were determined by two‐tailed Student's t‐test or via one‐way analysis of variance (ANOVA) with a Tukey post‐hoc test. A two‐tailed *P* < 0.05 was considered statistically significant.

## Conflict of Interest

The authors declare no conflict of interest.

## Supporting information



Supporting Information

## Data Availability

The data that support the findings of this study are available from the corresponding author upon reasonable request.
