# Supplementary material for: Artificial Intelligent‐Enhanced Metabolite Profiling for Intraoperative IDH1 Genotyping in Glioma Using an Orthogonally Responsive SERS Probe
Source: Adv Sci (Weinh). 2025 Apr 2;12(26):2503360. doi: 10.1002/advs.202503360 (PMC12245123; doi:10.1002/advs.202503360)
Supplement: Supplementary file 1 — Supporting Information [file ADVS-12-2503360-s001.docx]

Supporting Information

**Artificial Intelligent-Enhanced Metabolite Profiling for Intraoperative IDH1 Genotyping in Glioma Using an Orthogonally Responsive SERS Probe**

*Hang Yin, Xin Zhang, Zheng Zhao, Chong Cao, Minhua Xu, Suhongrui Zhou, Tian Xuan, Ziyi Jin, Limei Han, Yang Fan, Cong Wang*, Xiao Zhu*, Ying Mao*, Jinhua Yu*, Cong Li**

Supplementary Text

**Materials.** Dulbecco’s Modified Eagle’s Medium (DMEM), penicillin-streptomycin (PS), and fetal bovine serum (FBS) were obtained from Gibco (Waltham, USA). Phosphate buffered saline (PBS) and trypsin were purchased from Meilun Biotechnology (Dalian, China). HAuCl_4_·4H_2_O (99.95%, Au>47.8%) was purchased from HWRK Chemical Co., Ltd. AgNO_3_ (>99.8%) was purchased from Sinopharm Chemical Reagent Co., Ltd. Trisodium citrate dihydrate (AR,99.0%) was purchased from Aladdin Biochemical Technology Co., Ltd. L-Ascorbic acid (>98%) was purchased from Shanghai Yeasen BioTechnologies co., Ltd. Thiolated polyethylene glycol (SH-PEG, Mw ≈ 5000) was purchased from Beijing Jenkem Technology Company. Cell Counting Kit-8 (CCK-8) assay was obtained from Meilunbio®. Radionuclide ^99m^Tc were obtained from Huashan Hospital PET Center and Shanghai Cancer Center. Hydrogen peroxide test kit (S0038) and GSH and GSSG Assay Kit (S0053) were purchased from Beyotime.

**Synthesis of Raman reporter RR1.** Cy7-lip was synthesized following the published procedures by Malini Olivo's group.^[1]^ Cy7-lip (0.1 g, 0.14 mmol) and p-nitrothiophenol (0.042 g, 0.27 mmol) were dissolved in DCM (10 mL), and triethylamine (0.028 g, 0.28 mmol) was added. The reaction mixture was stirred under an argon atmosphere for 2 h at room temperature. The solvent was removed under reduced pressure, then the crude product was purified by column chromatography over silica gel [CH_2_Cl_2_: MeOH = 20:1 to 5:1, V:V] to afford a darkgreen solid RR1 (0.053 g, 44.5%). ^1^H NMR (400 MHz, CDCl_3_): δ 8.58 (d, *J* = 13.6 Hz, 2H), 8.13 (d, *J* = 7.9 Hz, 2H), 7.44 – 7.28 (m, 6H), 7.19 (dd, *J* = 20.4, 6.9 Hz, 4H), 6.32 (d, *J* = 14.1 Hz, 2H), 4.52 (t, 1H), 4.38 (m, 4H), 3.46 (m, 2H), 3.20 – 3.01 (m, 3H), 2.79 (m, 4H), 2.42 (d, *J* = 5.3 Hz, 2H), 2.11 (m, 2H), 1.85 (d, *J* = 5.2 Hz, 2H), 1.42 (d, *J* = 7.9 Hz, 12H), 1.25 (m, 8H). HRMS (ESI+): m/z calcd for [M-H]^+^ =850.3377, found 850.3369.

**Synthesis of Raman reporter RR2.** BC was synthesized following the published procedures by Lin's group.^[2]^ BC (0.1 g, 0.19 mmol), 4-Hydroxypiperidine (0.095 g, 0.94 mmol) and N,N-Diisopropylethylamine (DIEA, 0.074 g, 0.57 mmol) were dissolved in 20 ml of CH_2_Cl_2_ and stirred at room temperature for 10 min. Then Benzotriazole-1-yl-oxytripyrrolidinophosphonium hexafluorophosphate (PyBOP, 0.14 g, 0.27 mmol) was added and the reaction was carried out for another 2h at room temperature to obtain the compounds BCN (0.092 g, 78.0%). BCN was directly used in the next step without purification. BCN (0.092 g, 0.15 mmol), N,N’-Dicyclohexylcarbodiimide (DCC, 0.092 g, 0.45 mmol), 4-Dimethylaminopyridine (DMAP, 0.054 g, 0.44 mmol), and lipoic acid (0.15 g, 0.73 mmol) were dissolved in 10 ml of CH_2_Cl_2_ and stirred overnight at room temperature. Purification by silica gel column chromatography [CH_2_Cl_2_: MeOH = 20:1, V:V] provided pure compound RR2 (0.085 g, 70.2%) as a dark green solid. ^1^H NMR (600 MHz, MeOD) δ 9.10 (s, 1H), 8.25 (s, 1H), 7.72 (m, J = 27.7 Hz, 4H), 7.61 (m, J = 7.4 Hz, 2H), 7.34 (m, J = 8.5 Hz, 2H), 7.04 – 6.93 (m, 1H), 6.68 (d, J = 22.2 Hz, 1H), 4.60 (s, 1H), 3.77 (q, J = 7.2 Hz, 4H), 3.66 (q, J = 6.8 Hz, 4H), 3.50 (t, J = 7.4 Hz, 4H), 2.40 (t, J = 36.5 Hz, 2H), 2.04 (s, 1H), 1.91 (t, J = 42.3 Hz, 2H), 1.42 (m, 2H), 1.37 (m, J = 14.4, 7.3 Hz, 4H), 1.32 – 1.29 (m, 12H), 1.25 – 1.18 (m, 6H). HRMS (ESI+): m/z calcd for [M-H]^+^ =808.3449, found 808.3455.

**Electrostatic potential calculation parameters.** All-electron DFT calculations have been carried out by the latest version of ORCA quantum chemistry software (Version 6.0.0).^[3]^ For geometric optimization of ground-state structures, the corrected version of r2SCAN exchange-correlation functional proposed by Grimme (so-called r2SCAN-3c) was adopted.^[4]^ The singlet point energy calculations were performed with B3LYP functional and the def2-TZVP basis set.^[5]^ The SMD implicit solvation model was used to account for the solvation effect.^[6]^ The DFT-D3 dispersion correction with BJ-damping was applied to correct the weak interaction to improve the calculation accuracy.^[7]^ ESP analysis was performed by Multiwfn package.^[8]^

**Transmission electron microscopy (TEM).** For the observation of Au-R12P morphology and size, TEM imaging was performed using a JEOL 2100F (JEOL) field emission microscope. Sample aliquots were spotted onto a copper grid coated with amorphous carbon, and excess liquid was removed by wicking with filter paper. The grids were air-dried at room temperature. TEM images of Au-R12P were captured at 300 kV and processed with Digital Micrograph software (Version 1.71.38).

**Dynamic light scattering (DLS).** The size distributions and Zeta potential (ζ-potential) of Au-R12P were measured using a Malvern Zetasizer (ZS90, Malvern Instruments Ltd., UK) at 25 °C. The measurements were conducted in disposable Zeta potential cells. The ζ-potential was calculated using the Zetasizer software (Version 7.11) with the Smoluchowski model.

**Cellular uptake.** GL261 cells were seeded in six-well plates (1 × 10^5^ per well) and incubated until 85% confluency was achieved. The cells were then cultured with CY5-labeled Au-R12P in the DMEM medium at 37 °C for 4 h. Then cells were washed three times with PBS and analyzed by flow cytometry (BD, FACS Aria II). All data were analyzed with FlowJo 10 software (Tree Star Inc.).

**Bio-distribution studies.** IDH1-WT and IDH1-MUT glioma mouse models were intravenously injected with ^99m^Tc-Au-R12P (1.5−2.0 MBq). The mice were sacrificed at 0.5, 1, 2, 4, 8, 12, and 24 hours post-administration, and tissues (tumor, normal brain, heart, liver, spleen, lung, kidney, stomach, and intestine) were excised and weighed. Radioactivity was measured using a gamma counter (SN-684, Shanghai Hesuo Rihuan Photoelectric Instrument Co., Ltd., Shanghai, China). The decay-corrected radioactivity was determined, and the uptake of 99mTc-Au-R12P was represented as the percentage of the injected dose per gram of tissue (% ID/g).

**Population pharmacokinetics/pharmacodynamic (PopPKPD) model** **development.** A PopPKPD model was developed by nonlinear mixed-effects modeling software (NONMEM^[9]^, version 7.5, ICON Development Solutions, MD, USA) using first-order conditional estimation with interaction (FOCE-I) estimation method. Perl-speaks-NONMEM (PsN, version 5.2.6, Uppsala University, Sweden) and Pirana (version 3.0.0, Certara, USA) were used to facilitate the use of NONMEM. For diagnostic graphics, exploratory analyses, and post-processing of NONMEM outputs, R (version 4.3.2, R Foundation for Statistical Computing, Vienna, Austria) was employed. For radiation activity data in healthy mice plasma, one-, two- and three-compartment PK models were tested. Furthermore, based on the hypothetical probes distribution process from plasma to each tissue in mice, the radiological activity data of normal brain region or tumor in IDH1-MUT and IDH1-WT mice were modeled as shown in Figure 5c, where K_in_ denoted the rate constant of probes entering tumor or normal brain region from plasma, K_out_ denoted the elimination rate of probes from tumor or normal brain region. Kp was the tissue allocation coefficient and can be expressed as K_in_/K_out_. The model was selected based on the Akaike information criterion (AIC), objective function value (OFV), goodness-of-fit (GOF) plots and visual predictive check (VPC).

**SERS imaging time-window (S/N Ratio, PD) analysis.** The ratio of SERS intensity of tumor to normal brain (S/N ratio, PD) was described using an indirect pharmacodynamics model (Dayneka, Garg, & Jusko, 1993) as illustrated in Figure S25, Supporting Information. In this model, S_max_ ​represents the maximum promotion of the probes to the S/N ratio, while SC_50_​ is the concentration of probes required for half of this maximum promotion. K_in_ represents the zero-order production rate constant, and K_out_ ​denotes the first-order elimination rate constant. If the probe is not targeted, it is assumed that the tumor and normal brain region have the same amount of probe distribution, resulting in an S/N ratio of 1. This ratio was used as the baseline value of the effect compartment. The R package rxode2 (version 2.1.2) was employed to simulate the effect in each type of mouse model. When the S/N ratio exceeded 12, the corresponding time interval was considered the optimal surgical trial window.

**Scheme S1. Synthetic of the Raman reporter RR1.**

**Scheme S2. Synthetic of the Raman reporter RR2.**


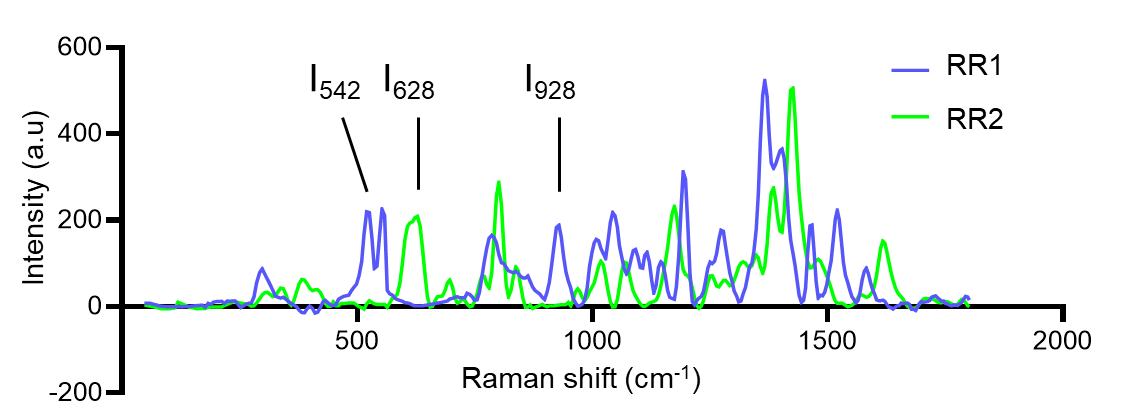


**Figure S1** Comparison of SERS spectra of RR1 and RR2.


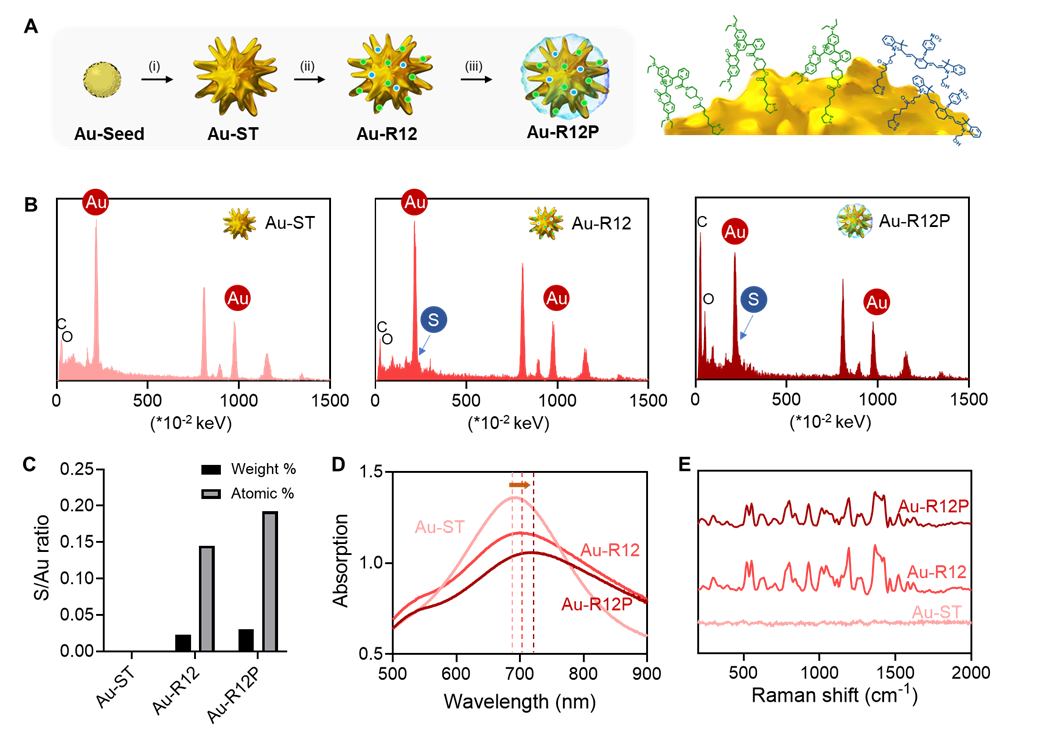


**Figure S2** (A) Synthetic procedure of Au-R12P. (B) Energy Dispersive Spectrometer (EDS) analysis of Au-ST, Au-R12, Au-R12P. (C) S/Au ratio of Au-ST, Au-R12, and Au-R12P. The "Weight %" group represents the relative weight ratio of sulfur to gold, while the "Atomic %" group represents the relative atomic ratio of sulfur to gold. (D) Absorption of Au-ST, Au-R12, Au-R12P in aqueous solution. (E) Raman spectra of Au-ST, Au-R12, Au-R12P.

**Figure S3** Raman spectra of Au-R12P at different concentrations (50 pM, 500 pM, 5.0 nM, 50 nM, 500 nM).


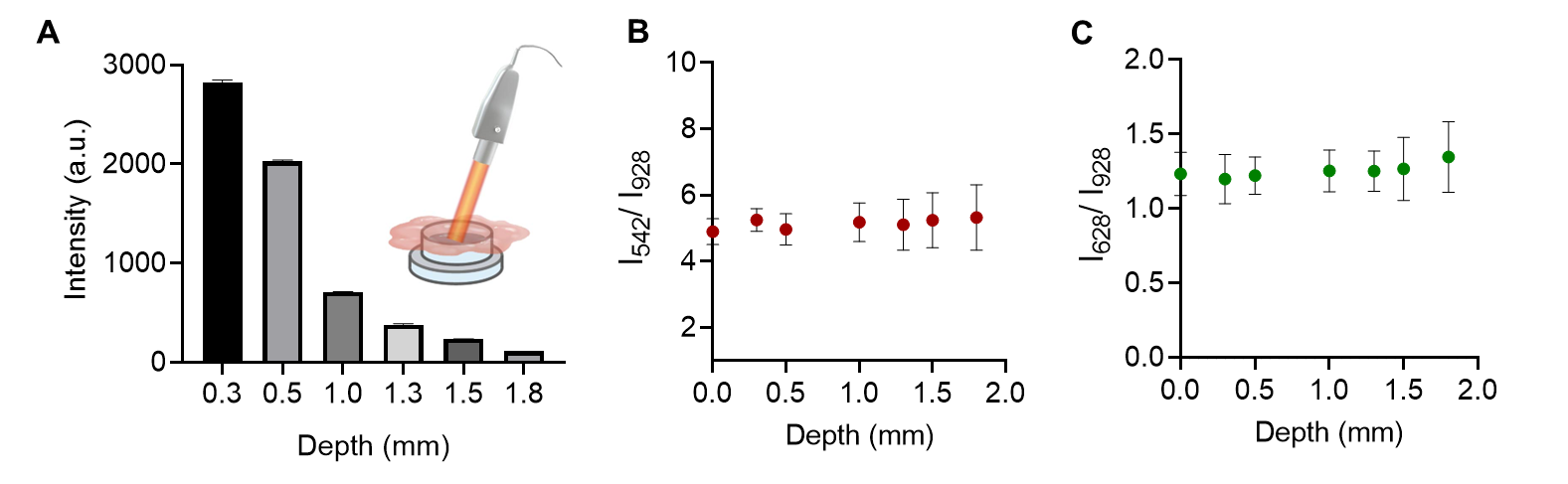


**Figure S4** Tissue thickness did not affect Raman ratios in resected tissues. (A) Raman signal intensities at 790 cm^-1^ of Au-R12P as a function of the thickness of covered mouse brain tissues. Data are raw spectra without de-baselining and presented as mean ± S.D. (*n* = 5). (B) Plots of the Raman intensity ratios (I_542_/I_928_) as a function of the thickness of covered mouse brain tissues. (*n* = 5). (C) Plots of the Raman intensity ratios (I_628_/I_928_) as a function of the thickness of covered mouse brain tissues. (*n* = 5).


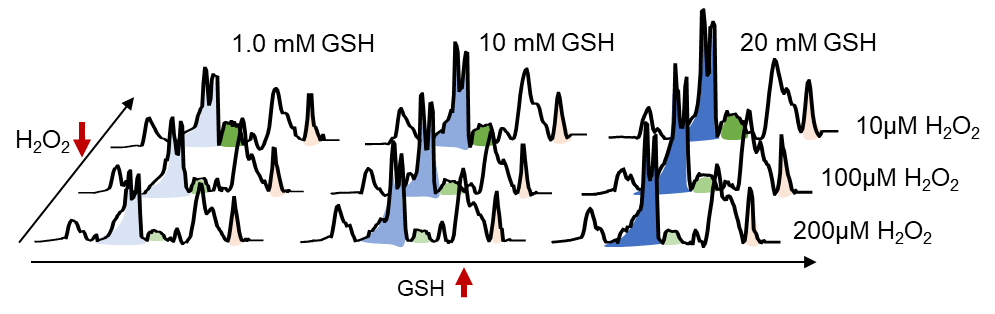


**Figure S5** Raman spectra of Au-R12P in mixtures comprising both H_2_O_2_ (10, 100, 200 μM) and GSH (1, 10, 20 mM).


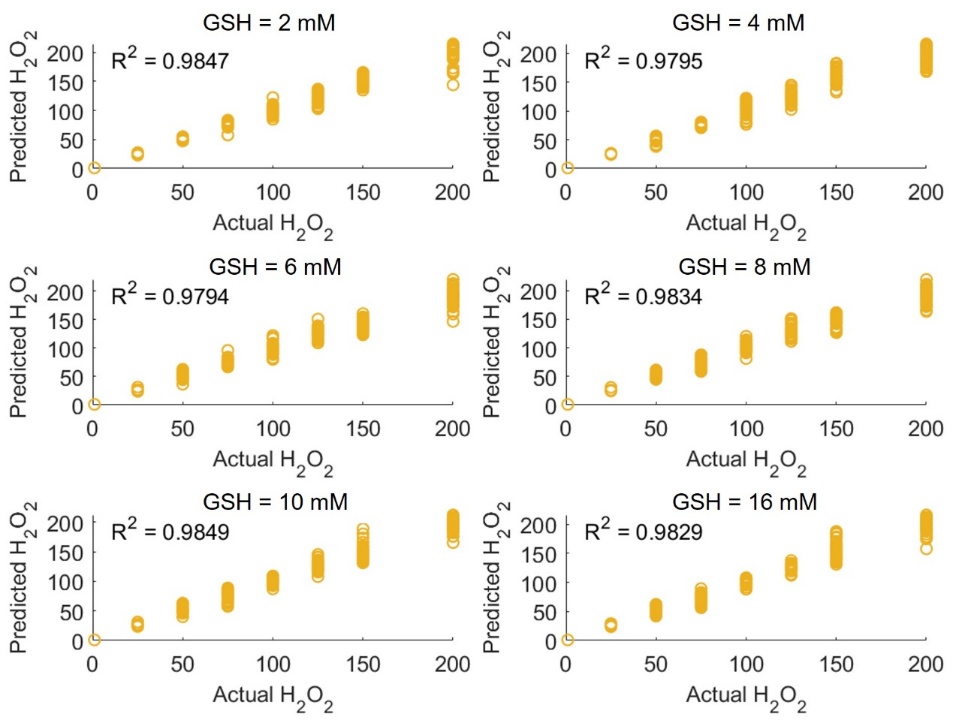


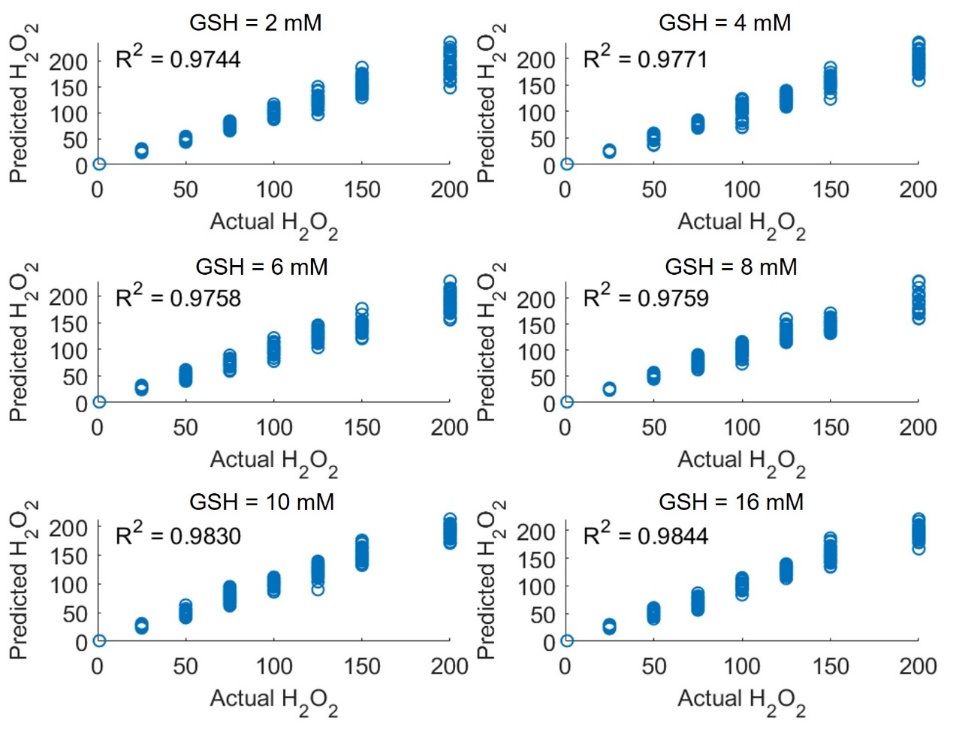


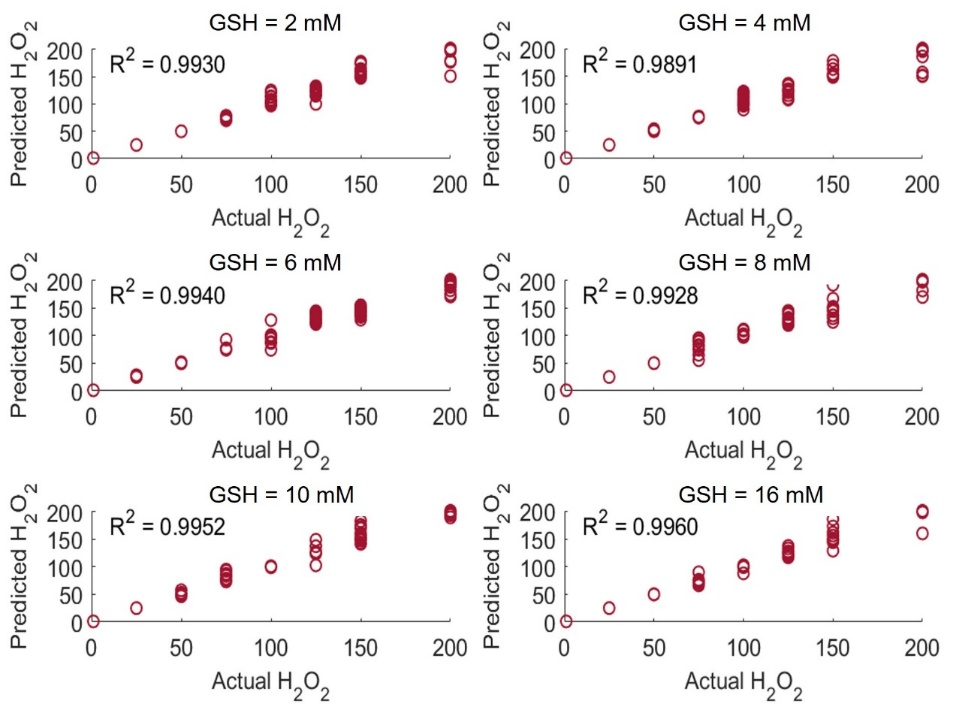


**Figure S6** Under the co-existence of different concentrations of GSH, H_2_O_2_ concentrations measured by 1D-ResNet (yellow), 2D-ResNet (blue), or DBCNet (red) algorithm. Each subgraph is displayed with the top 1% of outliers removed.


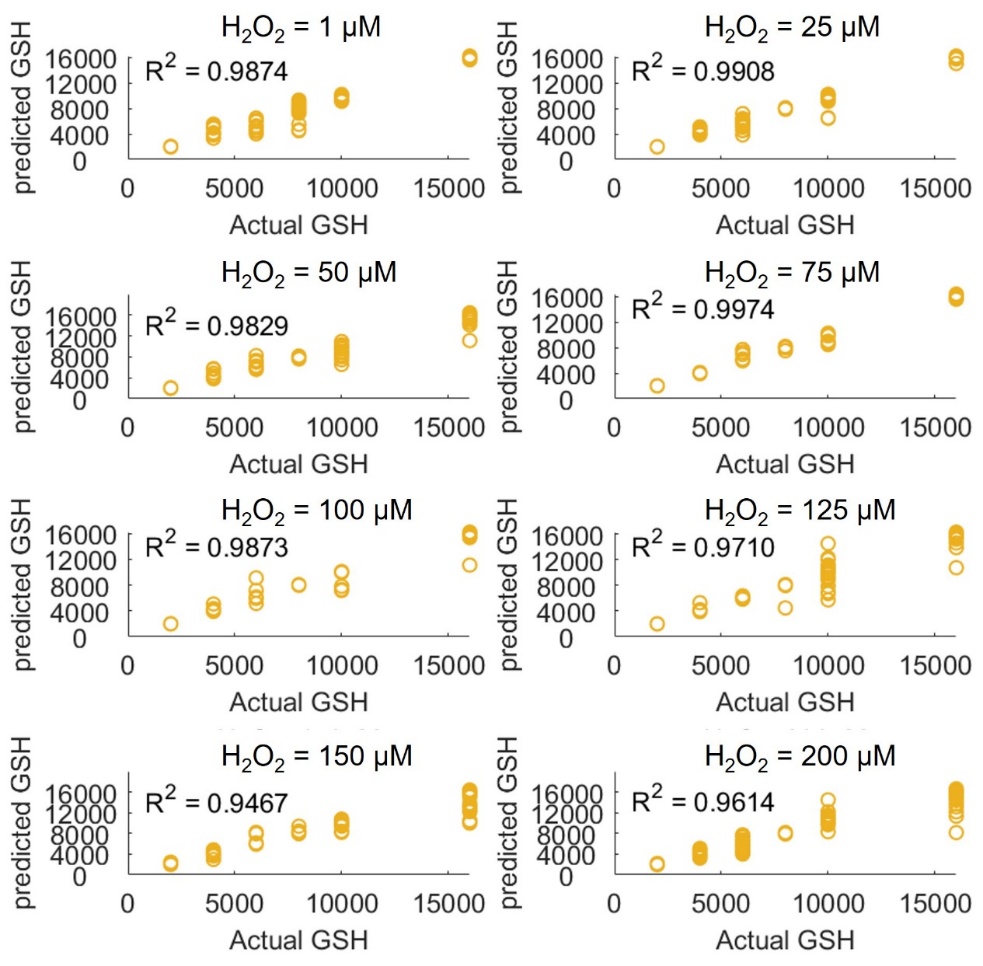


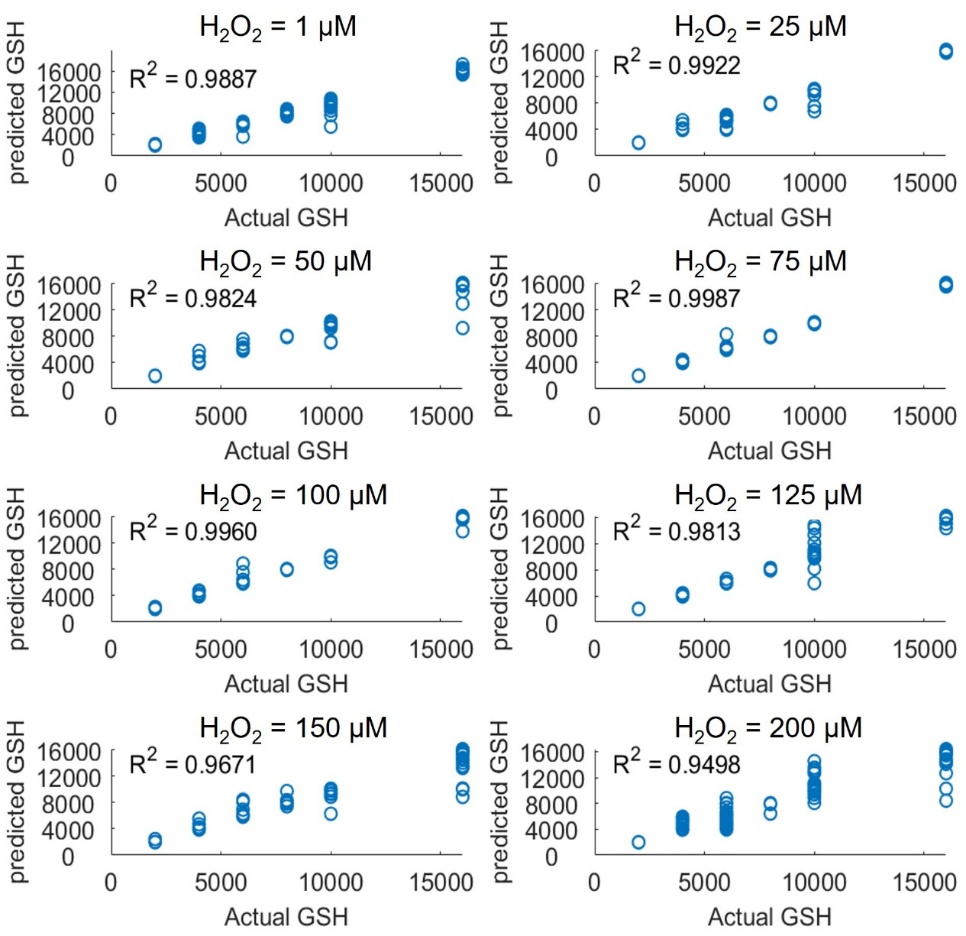


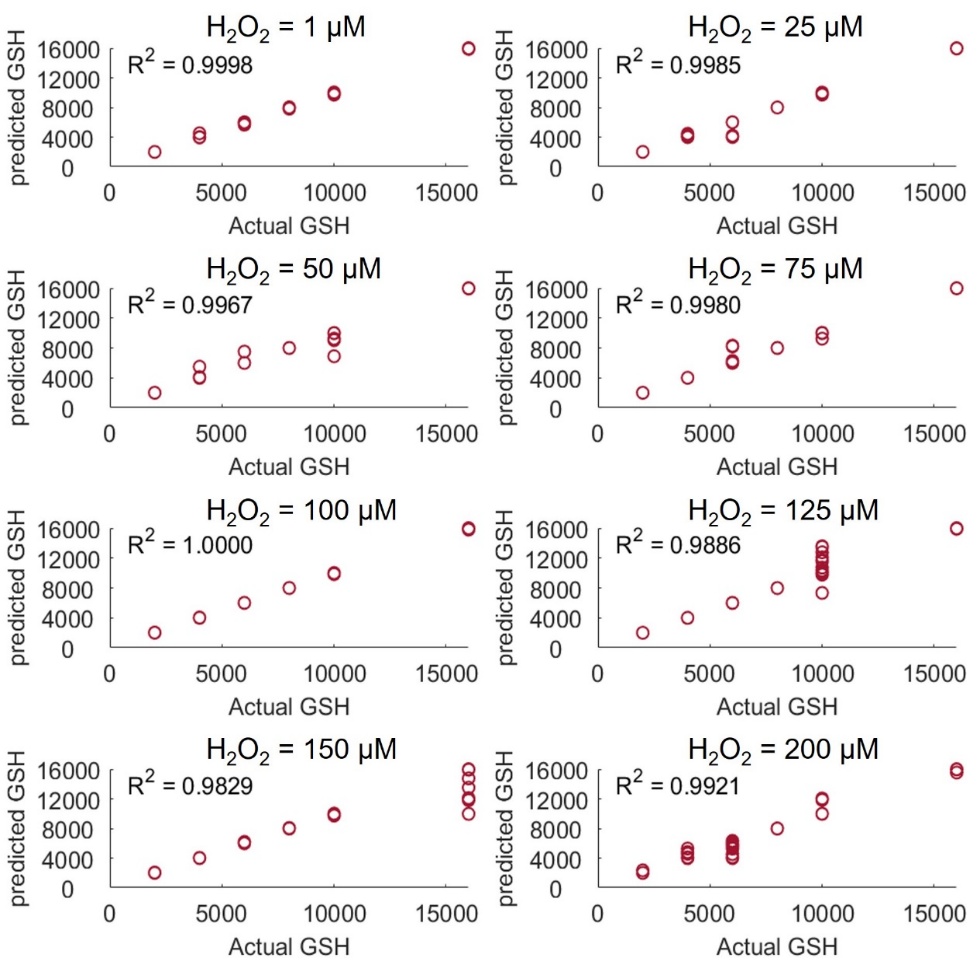


**Figure S7** Under the co-existence of different concentrations of H_2_O_2_, GSH concentrations measured by 1D-ResNet (yellow), 2D-ResNet (blue), or DBCNet (red) algorithm. Each subgraph is displayed with the top 1% of outliers removed.


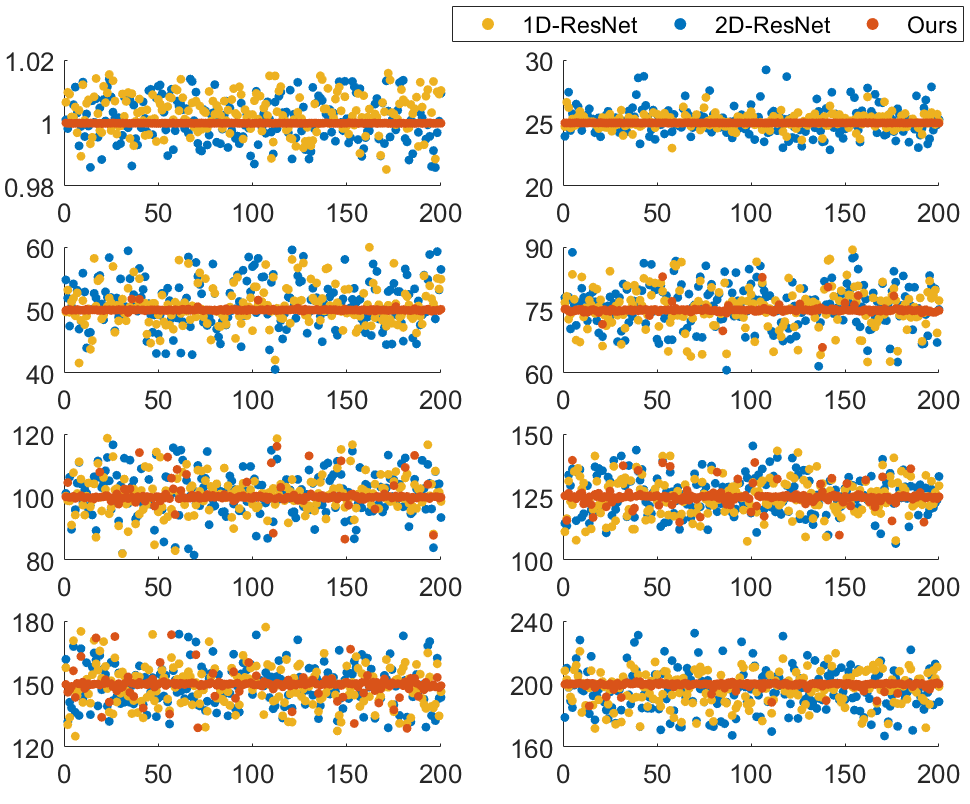


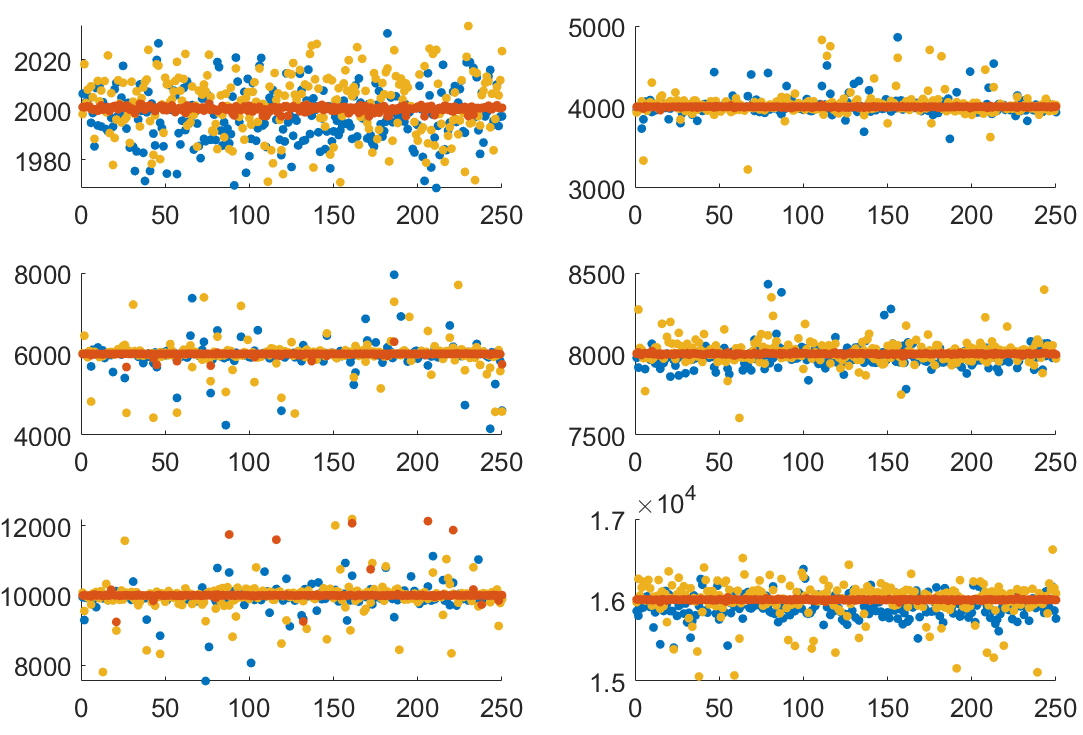


**Figure S8** The scatterplots of predicted GSH and H_2_O_2_ concentrations with assistance of 1D-ResNet, 2D-ResNet or DBCNet algorithms. Each subgraph is displayed with 200 and 250 randomly selected samples with top 5% of outliers removed for H_2_O_2_ and GSH in three models, respectively.

**
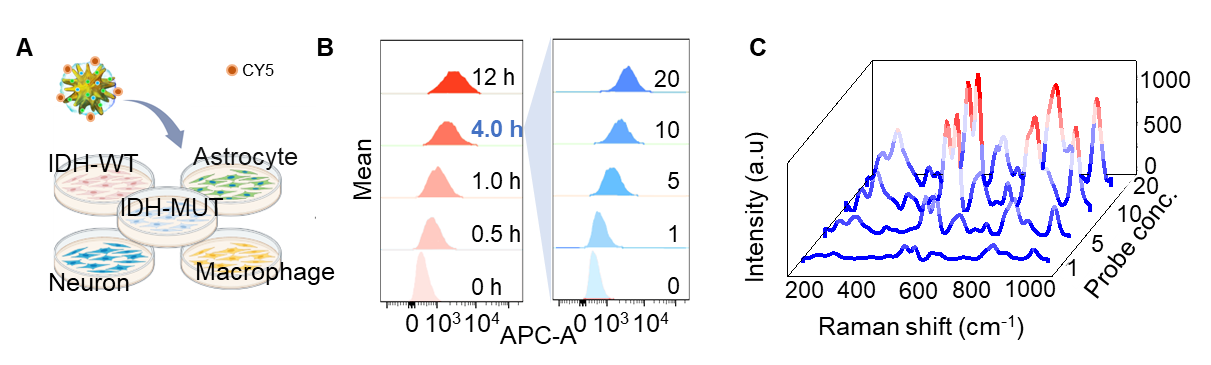
**

**Figure S9** (A) Schematic representation of probe uptake detection by both flow cytometry and Raman confocal microscopy. Created with BioRender.com. (B) Optimal time and dose for flow cytometric uptake of Au-R12P. (C) Characteristic SERS spectra of cells after incubation with different concentrations of Au-R12P for 4 hours.

**
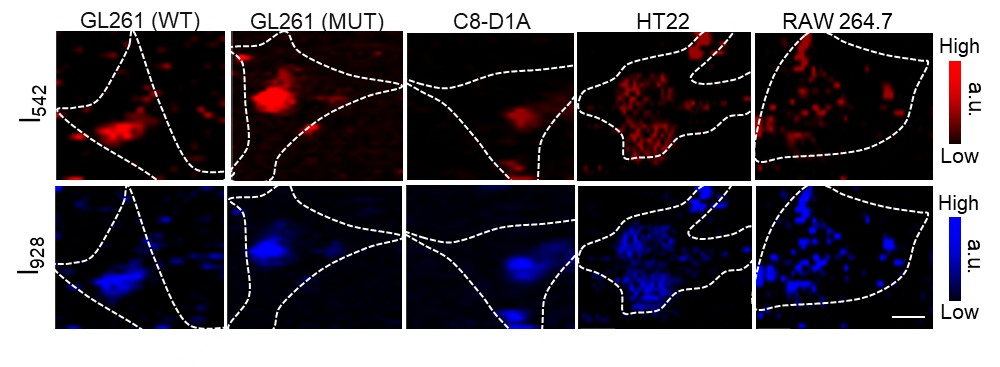
**

**Figure S10** Confocal Raman microscopic images of Au-R12P in above cells after ivosidenib (Ivo) treatment by collecting Raman signals at 542 cm^-1^ (upper) and 928 cm^-1^ respectively (lower). Scale bar = 10 μm.

**
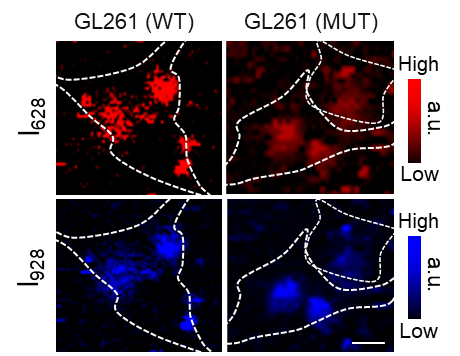
**

**Figure S11** Confocal Raman microscopic images of Au-R12P in live IDH1-WT and IDH1-MUT GL261 glioma cells by collecting Raman signals at 628 cm^-1^ (upper) and 928 cm^-1^ (lower) respectively. Scale bar = 10 μm.

**
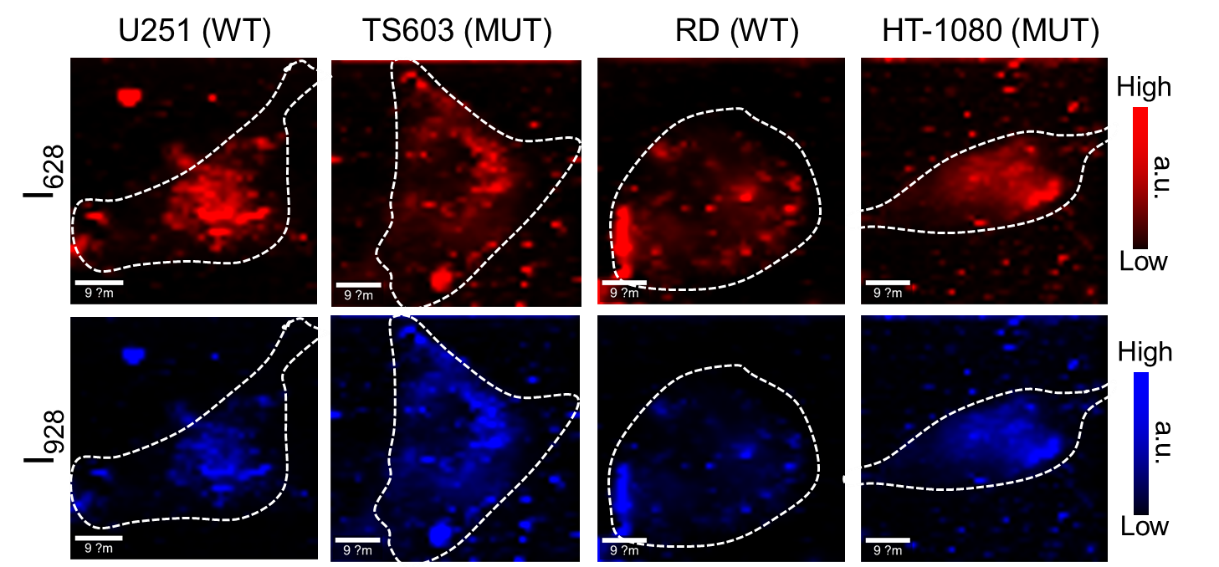
**

**Figure S12** Confocal Raman microscopic images of Au-R12P in cells (U251, TS603, RD, and HT-1080) by collecting Raman signals at 628 cm^-1^ (upper) and 928 cm^-1^ respectively (lower). Scale bar = 9 μm.

**
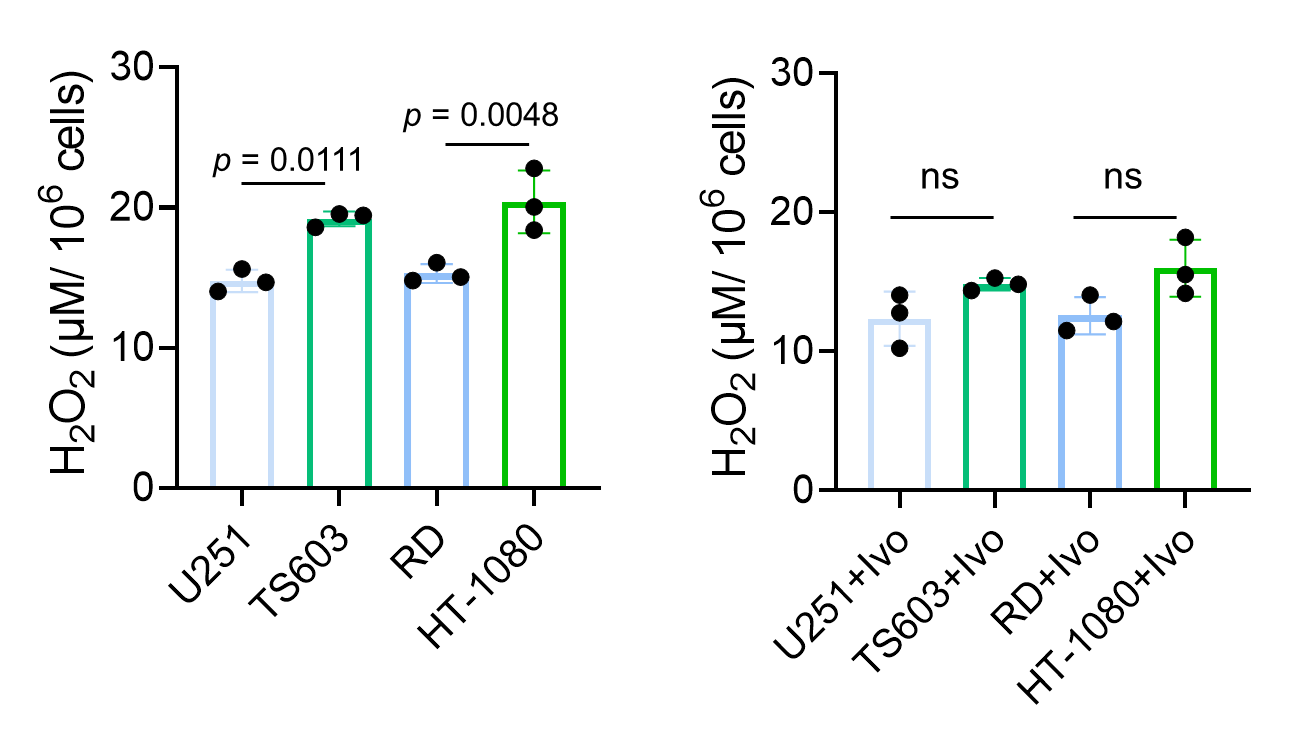
**

**Figure S13** H₂O₂ levels in cells before and after ivosidenib (Ivo) treatment, measured using a commercial detection kit.

**
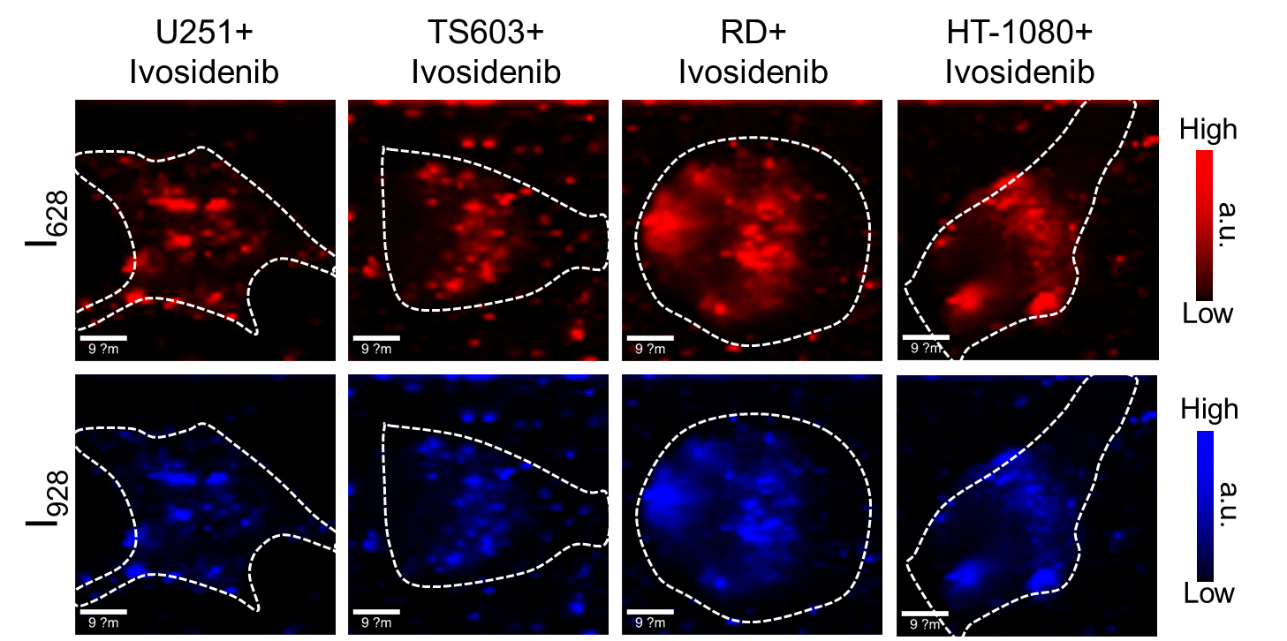
**

**Figure S14** Confocal Raman microscopic images of Au-R12P in cells after ivosidenib (Ivo) treatment by collecting Raman signals at 628 cm^-1^ (upper) and 928 cm^-1^ respectively (lower). Scale bar = 9 μm.

**
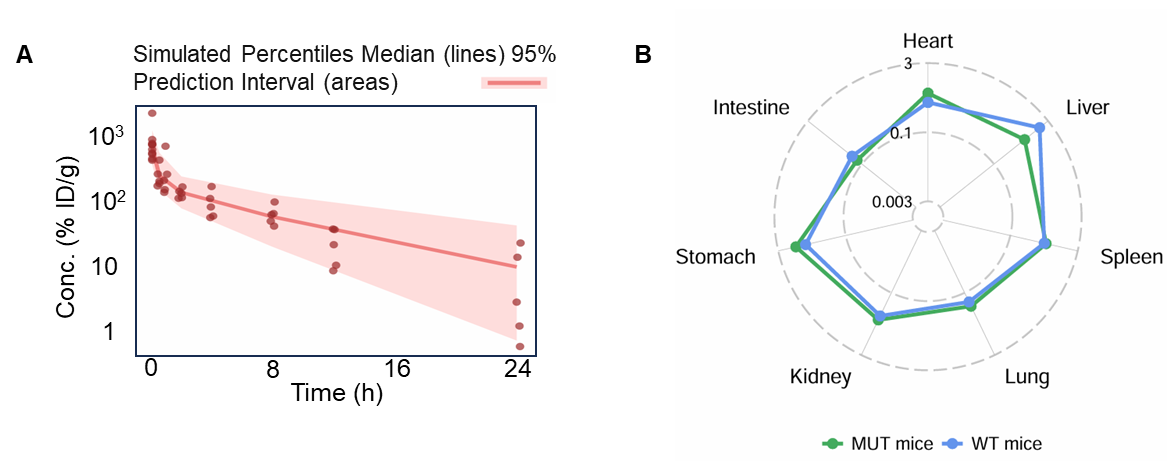
**

**Figure S15** (A) A visual predictive check from the PK model reveals the pharmacokinetic behavior of ^99m^Tc-Au-R12P in plasma that exhibits two-compartment disposition characteristics. The red dots are the experimental data, the solid red line indicates the predicted 50 percentile, and the light red area represents the 95% prediction interval of the final model simulation. (B) Partition coefficient (K_P_) of radioactive ^99m^Tc-Au-R12P distributing from the blood to the heart, liver, spleen, lung, kidney, stomach, and intestine after intravenous administration.


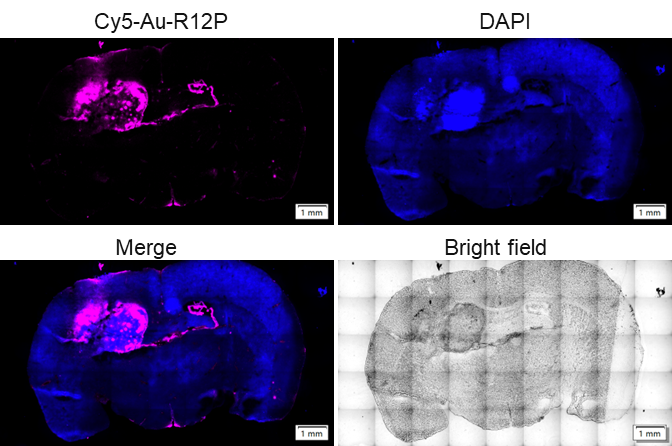


**Figure S16** Fluorescence confocal imaging of brain sections from tumor-bearing mice injected with CY5-labeled Au-R12P probes (1.50 nmol/g). Scale bar: 1 mm.

**
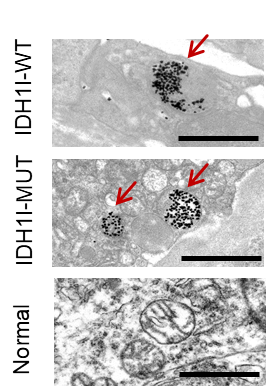
**

**Figure S17** TEM images of IDH1-WT/MUT GL261 glioma tissues at 8 h post intravenous injection of Au-R12P (1.50 nmol/g). Scale bar: 1.0 μm. ‘Normal’ refers to the normal brain tissue.


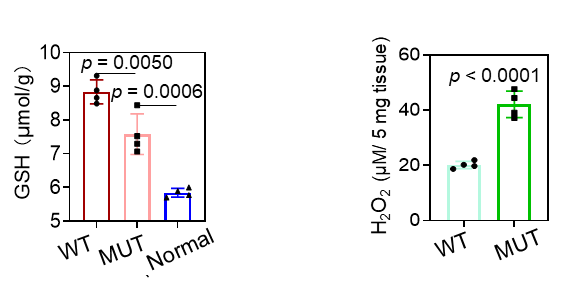


**Figure S18** Average GSH and H_2_O_2_ concentrations calculated by the commercial kits in tumor and contralateral normal brain tissue of mouse models (n = 4).


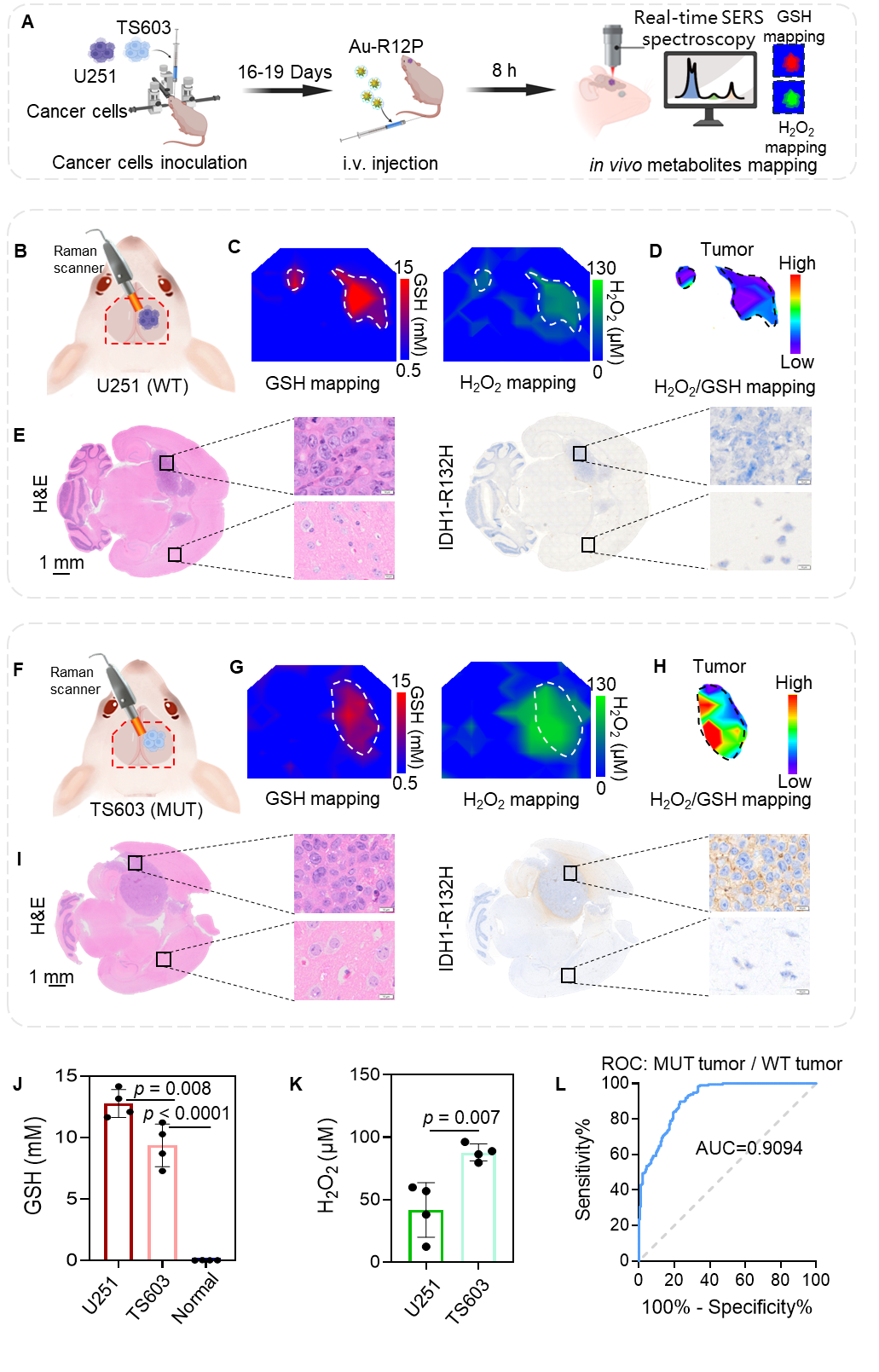


**Figure S19** Au-R12P successfully classified IDH genotypes of patient-derived IDH-mutant glioma xenografts in nude mouse models. (A) Schematic diagram of in vivo SERS imaging workflow. Au-R12P was injected in mouse models bearing U251 or TS603 glioma xenograft. Raman signal on the brain region was detected by a handheld Raman detector at 8 h post administration of the probe. Created with BioRender.com. (B) Diagram of intraoperative Raman imaging of U251 glioma xenograft during craniotomy. (C) In vivo GSH and H₂O₂ distribution maps of the exposed tumor region in U251 glioma models. (D) The distribution map of H₂O₂/GSH ratio in U251 tumor region at 8 h post probe administration. (E) Histopathologic H&E and IDH1-R132H immune-staining images of whole brain sections from mouse models bearing IDH1-WT glioma. (F) Diagram of intraoperative Raman imaging of TS603 glioma xenograft during craniotomy. (G) In vivo GSH and H₂O₂ distribution maps of the exposed tumor region in TS603 mutant glioma models. (H) The distribution map of H₂O₂/GSH ratio in TS603 tumor region at 8 h post probe administration. (I) Histopathologic H&E and IDH1-R132H immune-staining images of whole brain sections from mouse models bearing IDH1 mutant glioma. Average GSH (J) and H₂O₂ (K) concentrations in tumor and contralateral normal brain tissue (n = 4). (L) The results from the logistic regression model with I_628_/I_928_ ratios as explainable variables for the classification of different IDH1 genotypes. The ROC curve illustrates the performance of Au-R12P in discriminating IDH1 genotypes (right) in vivo.


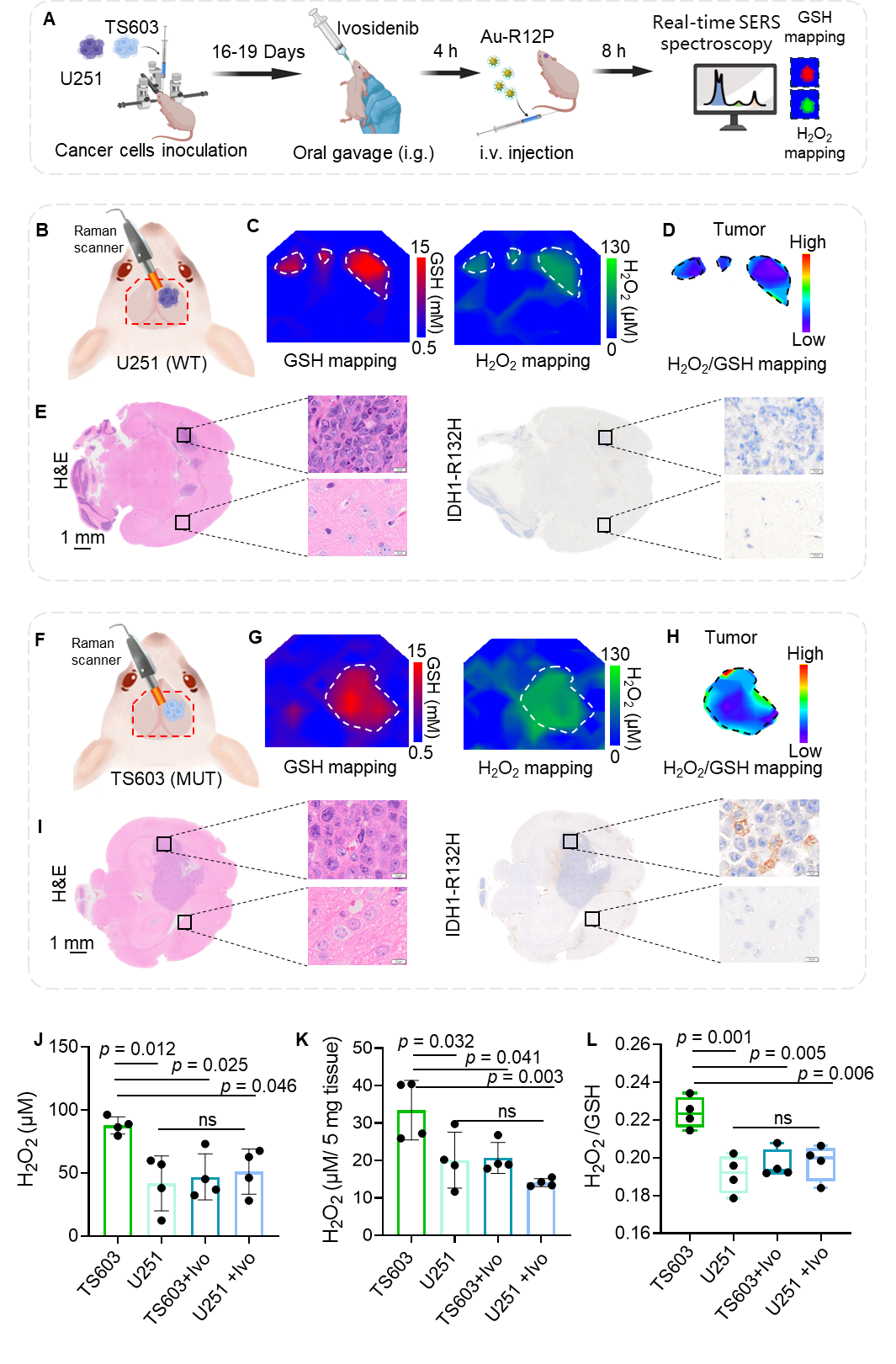


**Figure S20** Au-R12P signal correlated with IDH genotype-specific metabolic activity. (A) Schematic diagram of in vivo SERS imaging workflow. Ivosidenib (50 mg/kg) was orally administered to U251 and TS603 tumor-bearing nude mice. After 4 hours, Au-R12P was injected in mouse models bearing U251 or TS603 glioma xenograft. Raman signal on the brain region was detected by a handheld Raman detector at 8 h post administration of the probe. Created with BioRender.com. (B) Diagram of intraoperative Raman imaging of U251+ivosidenib glioma xenograft during craniotomy. (C) In vivo GSH and H₂O₂ distribution maps of the exposed tumor region in U251+ ivosidenib glioma models. (D) The distribution map of H₂O₂/GSH ratio in U251+ivosidenib tumor region at 8 h post probe administration. (E) Histopathologic H&E and IDH1-R132H immune-staining images of whole brain sections from mouse models bearing IDH1-WT glioma. (F) Diagram of intraoperative Raman imaging of TS603+ivosidenib glioma xenograft during craniotomy. (G) In vivo GSH and H₂O₂ distribution maps of the exposed tumor region in TS603+ivosidenib glioma models. (H) The distribution map of H₂O₂/GSH ratio in TS603+ivosidenib tumor region at 8 h post probe administration. (I) Histopathologic H&E and IDH1-R132H immune-staining images of whole brain sections from mouse models bearing IDH1 mutant glioma. (J) Average H₂O₂ concentrations in tumor tissue (n = 4). (K) H₂O₂ levels in TS603, U251, TS603+ivosidenib, U251+ivosidenib tumor tissues were measured by commercial kits (n = 4). (L) H₂O₂/GSH ratio of TS603, U251, TS603+ivosidenib, U251+ivosidenib tumor tissues (n = 4).


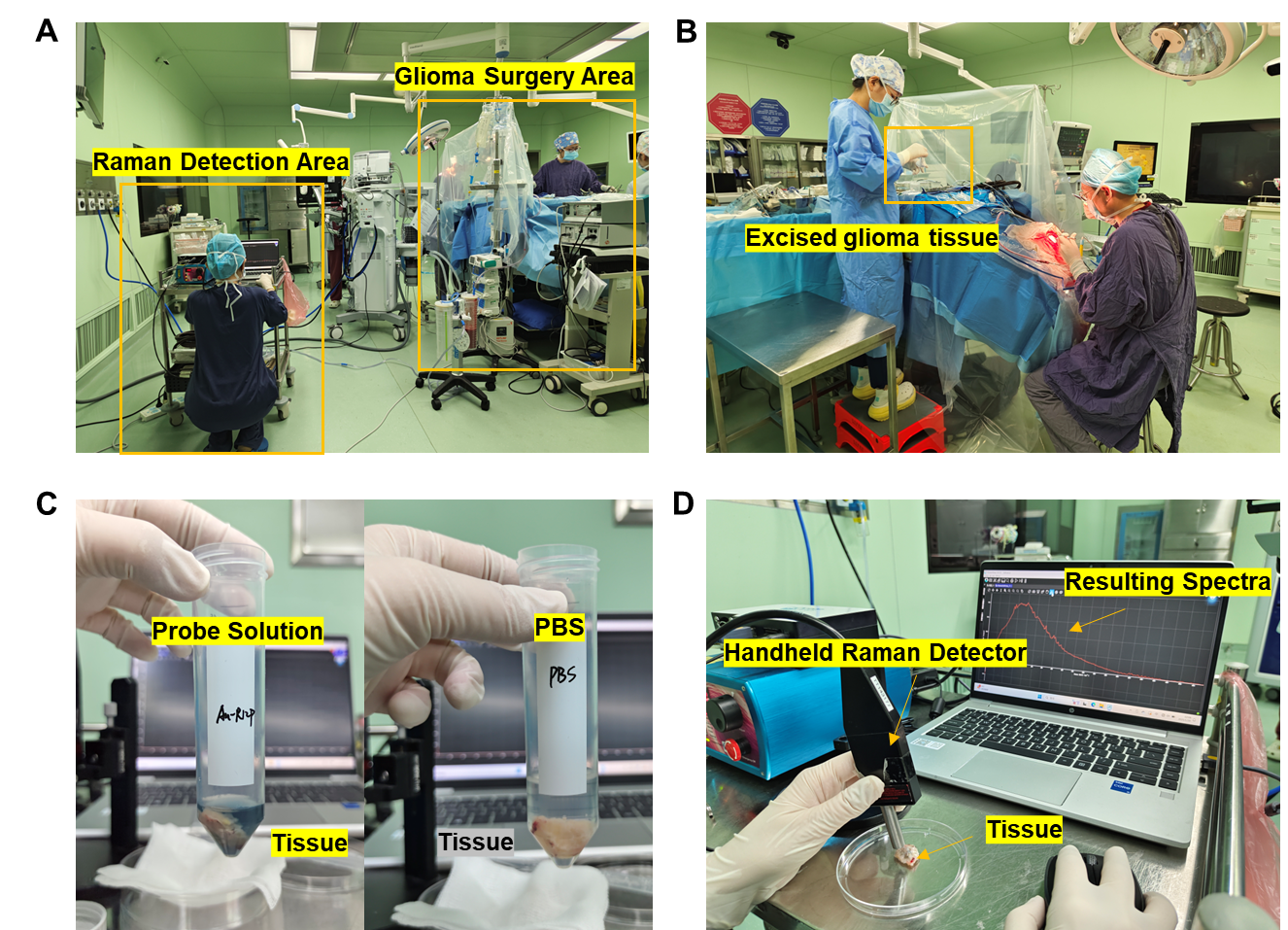


**Figure S21** Intraoperative workflow for tumor tissue analysis using Raman spectroscopy. (A) Setup of Raman equipment in the operating room. (B) Transfer of excised tumor tissue to the experimental personnel. (C) Incubation of tumor tissue in probe solution for 5 minutes followed by washing with PBS (D) Acquisition of Raman signal from tumor tissue and processing the spectra. The clinical research protocol was approved by the Ethics Committee of Huashan Hospital of Fudan University (No.603, 2023).


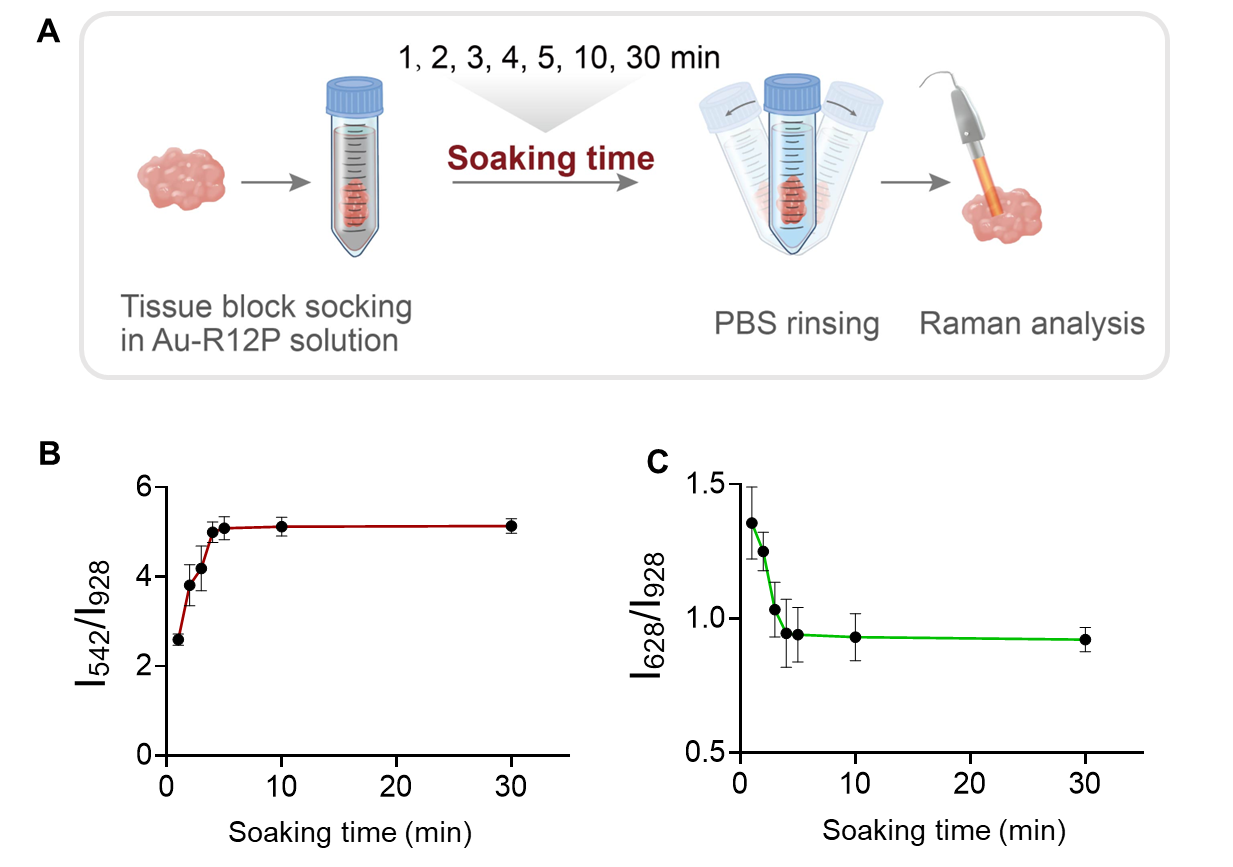


**Figure S22** (A) Schematic representation of the probe immersed for different times (1, 2, 3, 4, 5, 10, 30 min). (B, C) Curves of I_542_/I_928_ and I_628_/I_928_ with probe immersion time in glioma tissues. n = 3.


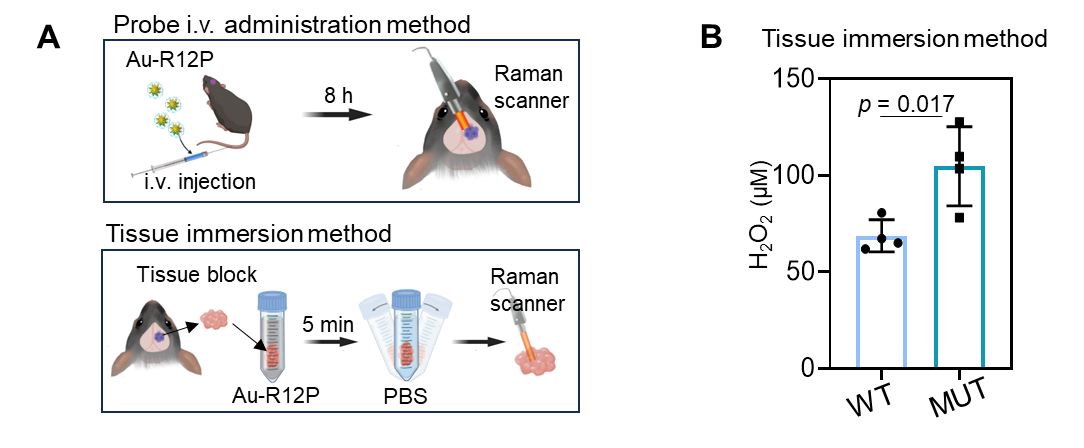


**Figure S23** (A) Schematic illustration of the probe i.v. administration method and tissue immersion method. (B) Average H₂O₂ concentrations measured using the tissue immersion-based method in IDH1-WT/MUT tumor tissue (n = 4). The probe solution was prepared at a concentration of 20 μM. Immediately after tissue excision, the samples were immersed in the solution for 5 minutes, followed by three washes with PBS before Raman signal detection. The results of the probe i.v. administration method are shown in Figure 6K of the main text.


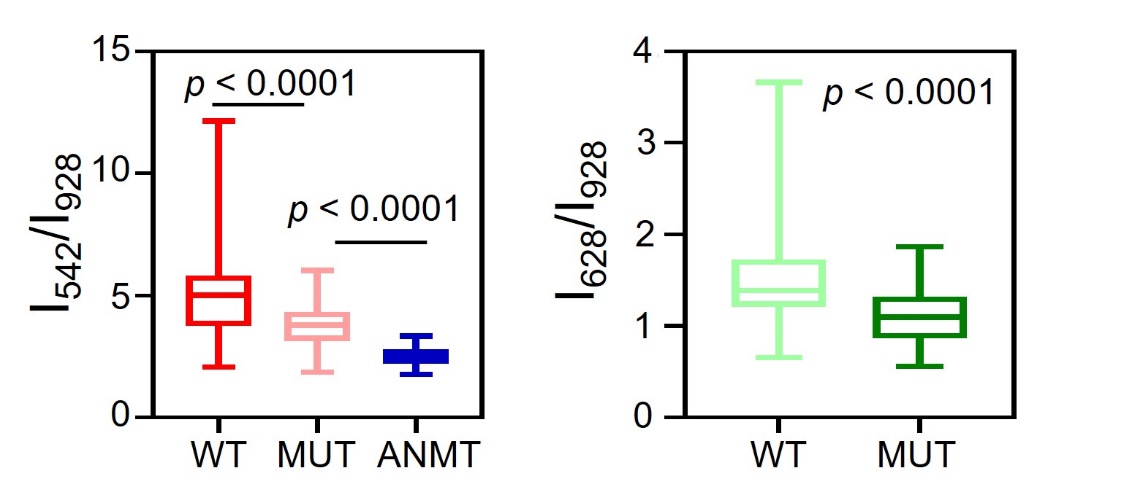


**Figure S24** I_542_/I_928_ ratios in tumor and adjacent non-malignant tissues (left). I_628_/I_928_ ratios in IDH1-WT and IDH1-MUT glioma tissues (right). Higher I_542_/I_928_ ratios represent higher concentrations of GSH, and lower I_628_/I_928_ ratios represent higher concentrations of H_2_O_2_.WT: IDH1-wild type glioma; MUT: IDH1-mutant glioma; ANMT: Adjacent non-malignant tissues.


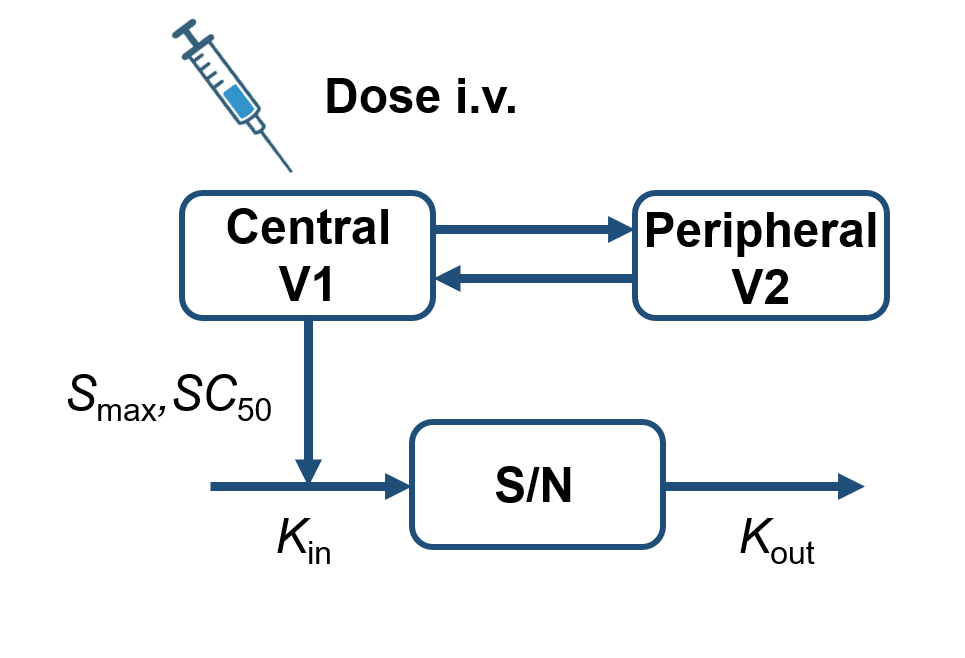


**Figure S25** Schematic of the PKPD model of Au-R12P. The final PKPD model consisted of a two-compartment plasma PK model along with an indirect effect compartment for S/N of the probes. Created with BioRender.com.

**
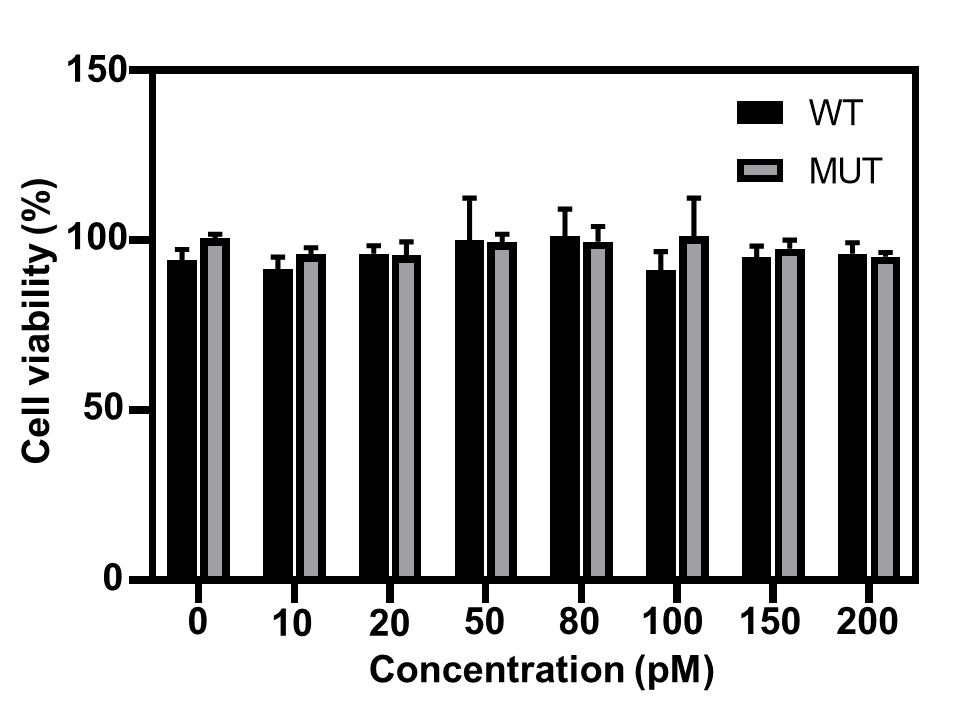
**

**Figure S26** Au-R12P shows minimized cytotoxicity. Viabilities of GL261-wild type (WT) and GL261-mutant (MUT) after treatment of Au-R12P for 48 h with concentrations ranging from 0 to 200 pM. Cell viabilities were determined by CCK-8 assay. Data are presented as the mean ± s.d. for three replicates.


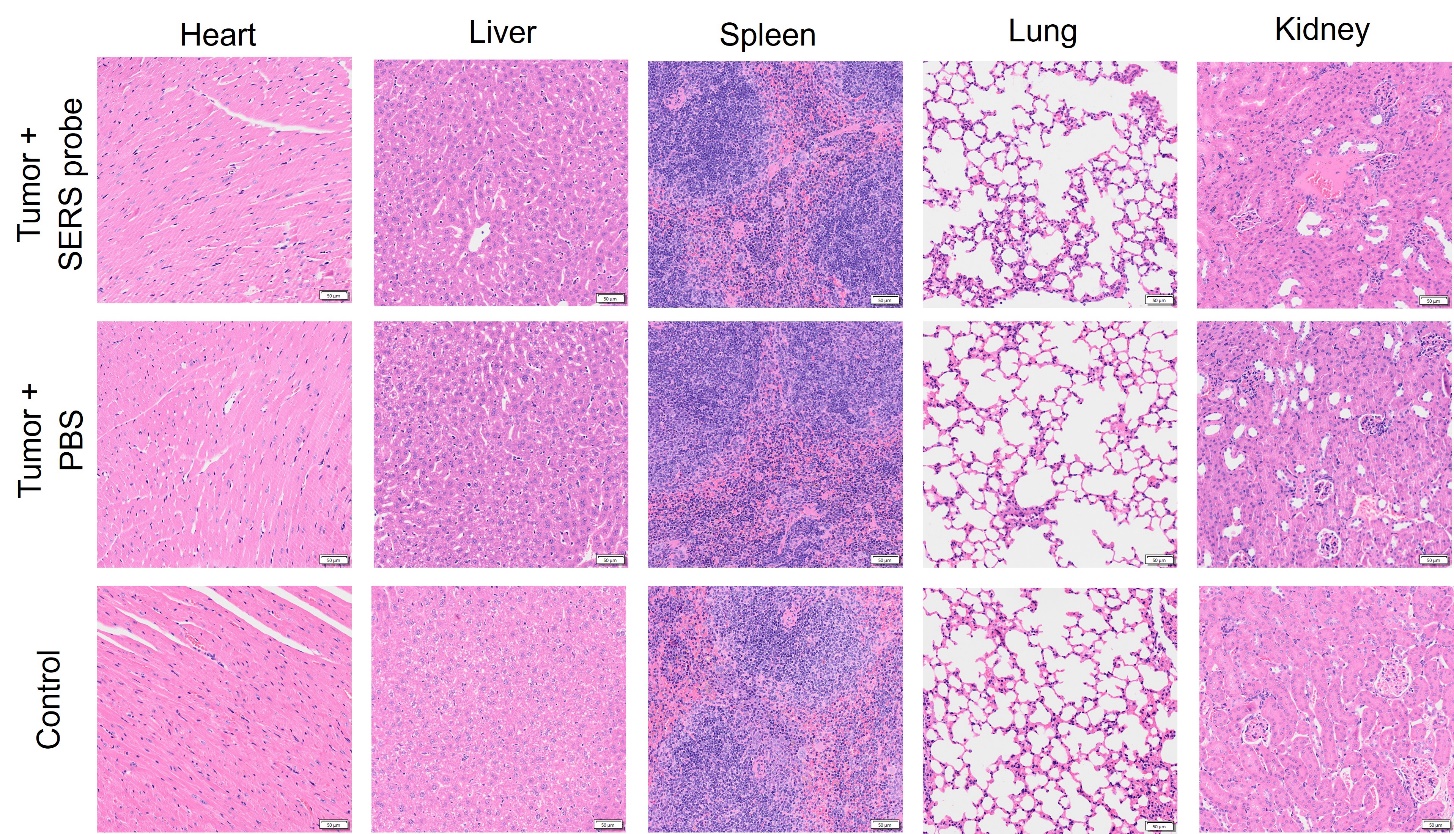


**Figure S27** Immuno-histological staining images of mouse major organs (heart, liver, spleen, lung, and spleen) from different groups. Scale bar = 50 μm.

Table S1.

Predicted concentrations of GSH determined by 1D-ResNet, 2D-ResNet or DBCNet algorithm.

| GSH ($\mu M$) | MAE | | | SD | | | RMSE | | |
| --- | --- | --- | --- | --- | --- | --- | --- | --- | --- |
|  | 1D-ResNet | 2D-ResNet | DBC  Net | 1D-ResNet | 2D-ResNet | DBC  Net | 1D-ResNet | 2D-ResNet | DBC  Net |
| 2000 | 53 | 33 | 2 | 425 | 337 | 20 | 428 | 338 | 20 |
| 4000 | 145 | 149 | 46 | 300 | 379 | 241 | 333 | 406 | 245 |
| 6000 | 347 | 350 | 123 | 856 | 822 | 516 | 922 | 892 | 530 |
| 8000 | 135 | 122 | 19 | 405 | 559 | 241 | 426 | 571 | 241 |
| 10000 | 422 | 381 | 279 | 938 | 951 | 1039 | 1027 | 1023 | 1074 |
| 16000 | 561 | 379 | 94 | 1465 | 1130 | 635 | 1566 | 1191 | 641 |
| Average | 282 | 240 | 96 | 863 | 774 | 567 | 908 | 810 | 575 |

Table S2.

Predicted concentrations of H_2_O_2_ determined by 1D-ResNet, 2D-ResNet or DBCNet algorithm.

| H_2_O_2_ ($\mu M$) | MAE | | | SD | | | RMSE | | |
| --- | --- | --- | --- | --- | --- | --- | --- | --- | --- |
|  | 1D-ResNet | 2D-ResNet | DBCNet | 1D-ResNet | 2D-ResNet | DBCNet | 1D-ResNet | 2D-ResNet | DBCNet |
| 1 | 0.01 | 0.01 | 0.00 | 0.01 | 0.00 | 0.00 | 0.01 | 0.01 | 0.00 |
| 25 | 0.82 | 1.28 | 0.02 | 2.14 | 2.02 | 0.20 | 2.29 | 2.39 | 0.21 |
| 50 | 3.22 | 4.03 | 0.94 | 3.76 | 4.52 | 4.47 | 4.94 | 6.04 | 4.56 |
| 75 | 5.00 | 5.58 | 1.49 | 4.96 | 5.07 | 4.78 | 7.04 | 7.53 | 5.00 |
| 100 | 5.50 | 6.99 | 2.64 | 5.56 | 6.48 | 6.01 | 7.81 | 9.52 | 6.55 |
| 125 | 6.72 | 7.53 | 2.97 | 5.59 | 6.17 | 5.88 | 8.74 | 9.72 | 6.57 |
| 150 | 9.69 | 9.35 | 4.52 | 9.17 | 7.89 | 9.04 | 13.33 | 12.22 | 10.09 |
| 200 | 11.20 | 13.64 | 2.85 | 11.64 | 12.15 | 8.81 | 16.14 | 18.25 | 9.24 |
| Average | 5.20 | 5.96 | 1.90 | 7.30 | 7.62 | 5.98 | 8.96 | 9.67 | 6.28 |

Table S3.

Demographic and clinical characteristics of Asian patients.

| NO. | Gender | Age | WHO grade | Pathological  classification | IDH classification |
| --- | --- | --- | --- | --- | --- |
| 1 | Female | 57 | IV | Glioblastoma | IDH1-WT |
| 2 | Female | 54 | IV | Glioblastoma | IDH1-WT |
| 3 | Female | 35 | IV | Glioblastoma | IDH1-WT |
| 4 | Female | 54 | IV | Glioblastoma | IDH1-WT |
| 5 | Female | 45 | IV | Glioblastoma | IDH1-WT |
| 6 | Male | 76 | IV | Glioblastoma | IDH1-WT |
| 7 | Male | 53 | IV | Glioblastoma | IDH1-WT |
| 8 | Male | 22 | IV | Glioblastoma | IDH1-WT |
| 9 | Male | 65 | IV | Glioblastoma | IDH1-WT |
| 10 | Male | 28 | IV | Glioblastoma | IDH1-WT |
| 11 | Male | 67 | IV | Glioblastoma | IDH1-WT |
| 12 | Male | 52 | IV | Glioblastoma | IDH1-WT |
| 13 | Male | 70 | IV | Glioblastoma | IDH1-WT |
| 14 | Male | 38 | II | Astrocytoma | IDH1-WT |
| 15 | Male | 59 | II | Astrocytoma | IDH1-WT |
| 16 | Female | 52 | II | Astrocytoma | IDH1-WT |
| 17 | Female | 21 | III | Astrocytoma | IDH1-WT |
| 18 | Male | 42 | III | Astrocytoma | IDH1-WT |
| 19 | Male | 39 | II | Astrocytoma | IDH1-MUT |
| 20 | Male | 53 | II | Astrocytoma | IDH1-MUT |
| 21 | Female | 60 | II | Astrocytoma | IDH1-MUT |
| 22 | Female | 26 | III | Astrocytoma | IDH1-MUT |
| 23 | Male | 36 | III | Astrocytoma | IDH1-MUT |
| 24 | Female | 55 | IV | Astrocytoma | IDH1-MUT |
| 25 | Male | 41 | IV | Astrocytoma | IDH1-MUT |
| 26 | Male | 48 | II | Oligodendroglioma | IDH1-MUT |
| 27 | Male | 51 | II | Oligodendroglioma | IDH1-MUT |
| 28 | Male | 48 | II | Oligodendroglioma | IDH1-MUT |
| 29 | Male | 34 | II | Oligodendroglioma | IDH1-MUT |
| 30 | Male | 33 | II | Oligodendroglioma | IDH1-MUT |
| 31 | Female | 57 | II | Oligodendroglioma | IDH1-MUT |

**NMR and MS spectrum data**

**
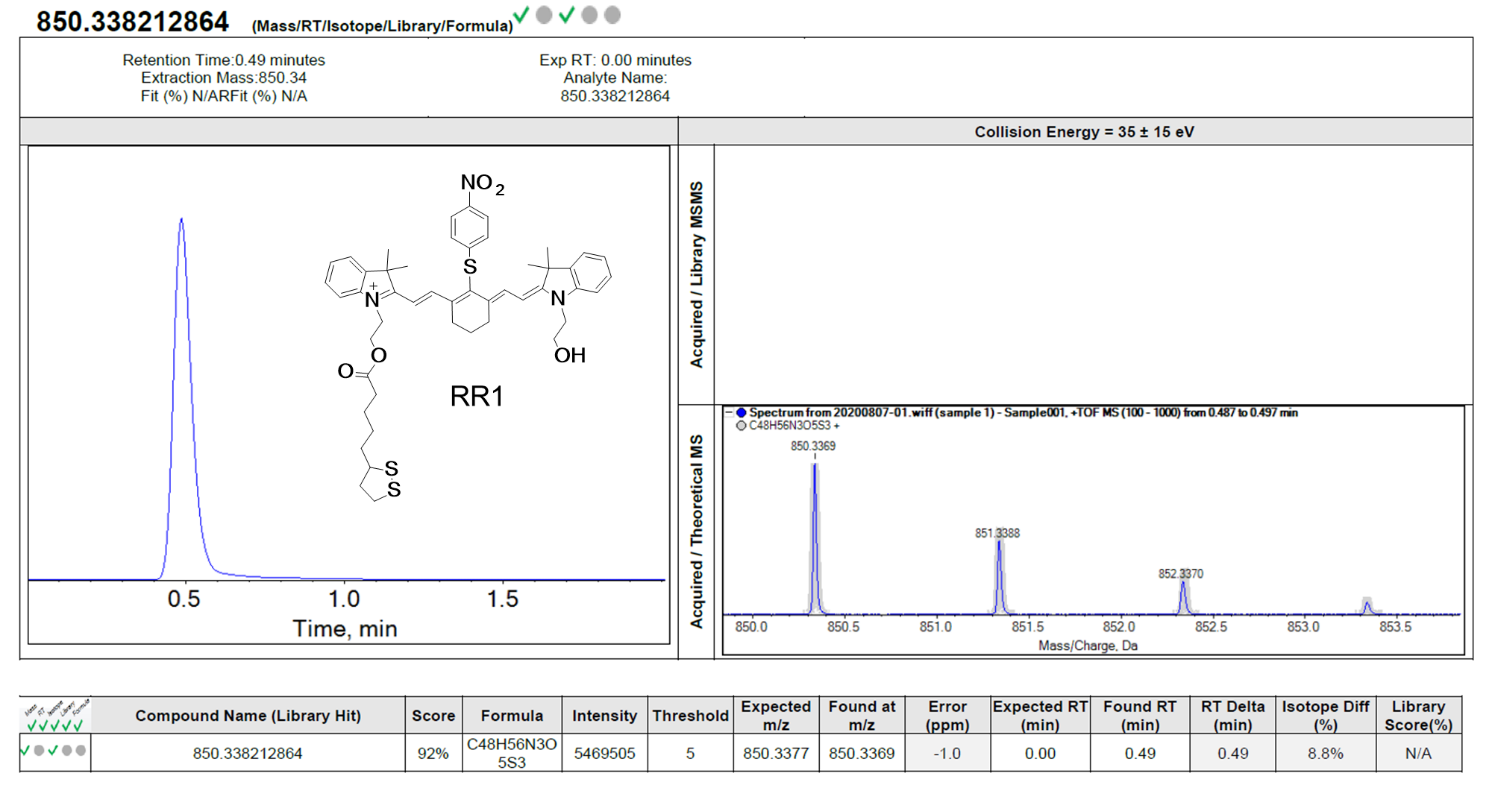
**

**Data S1. High-resolution mass spectrometry (HR-MS) of RR1.**

**
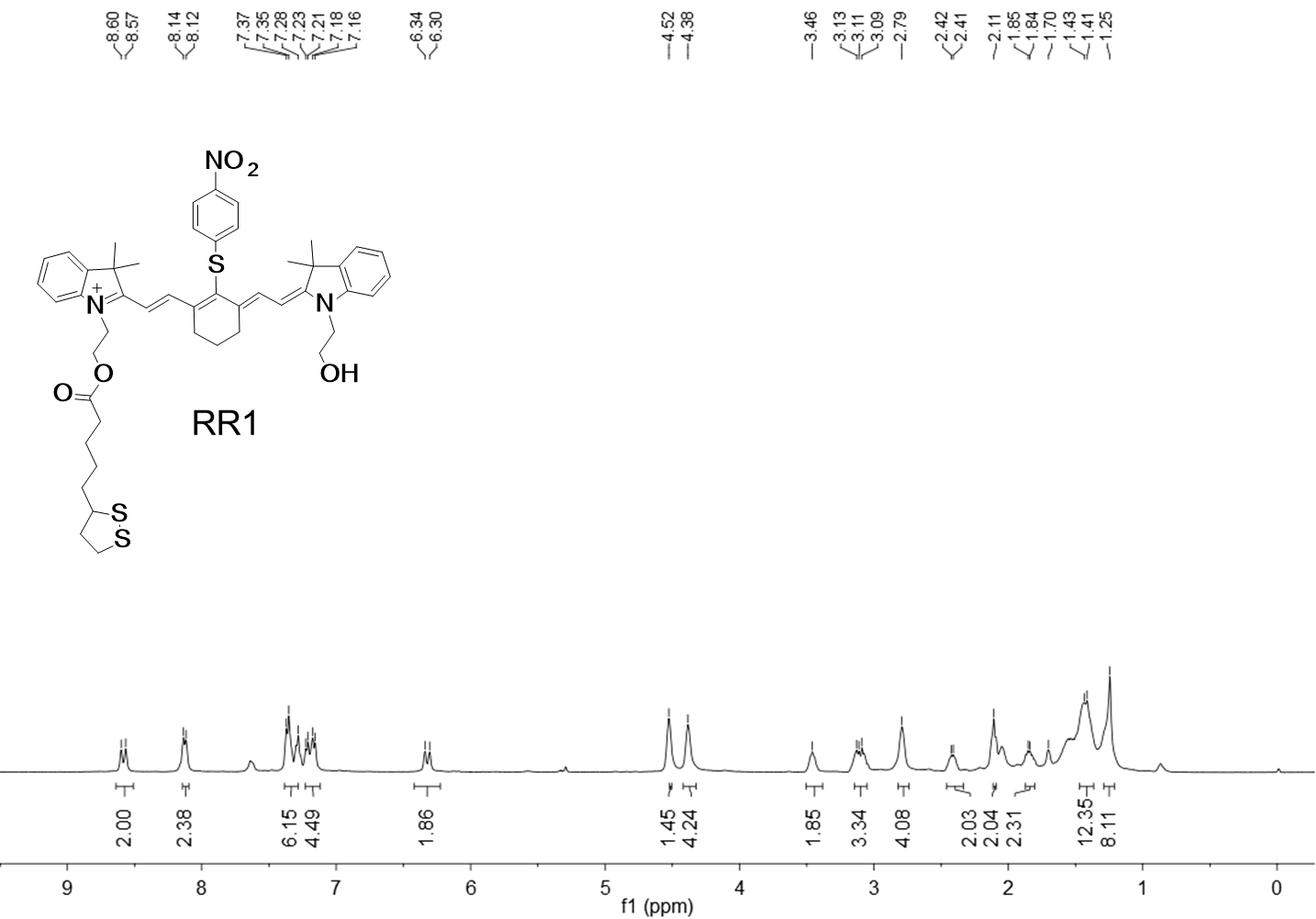
**

**Data S2. ^1^H NMR spectrum of RR1 in DMSO-d6.**

**
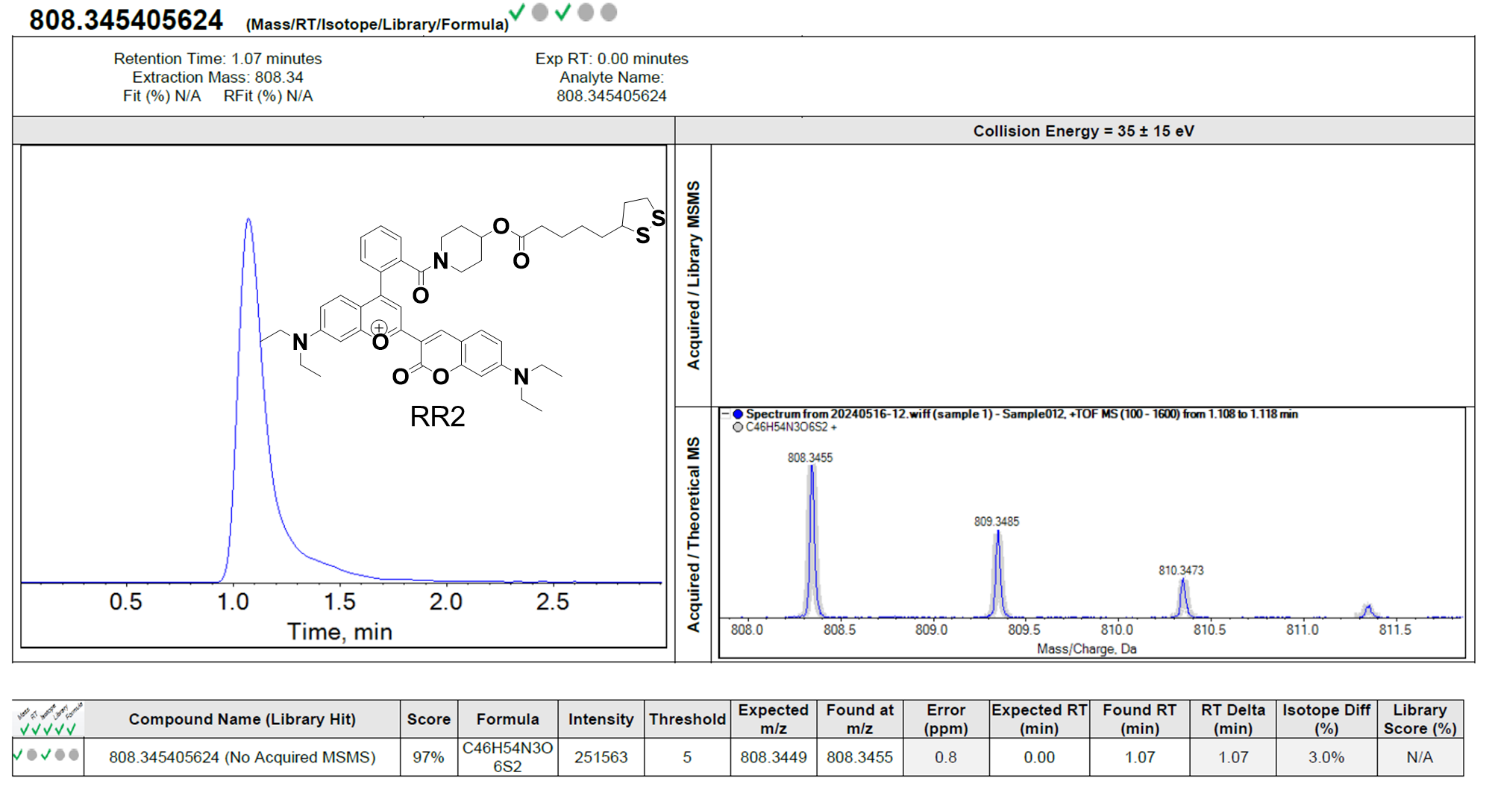
**

**Data S3. High-resolution mass spectrometry (HR-MS) of RR2.**

**
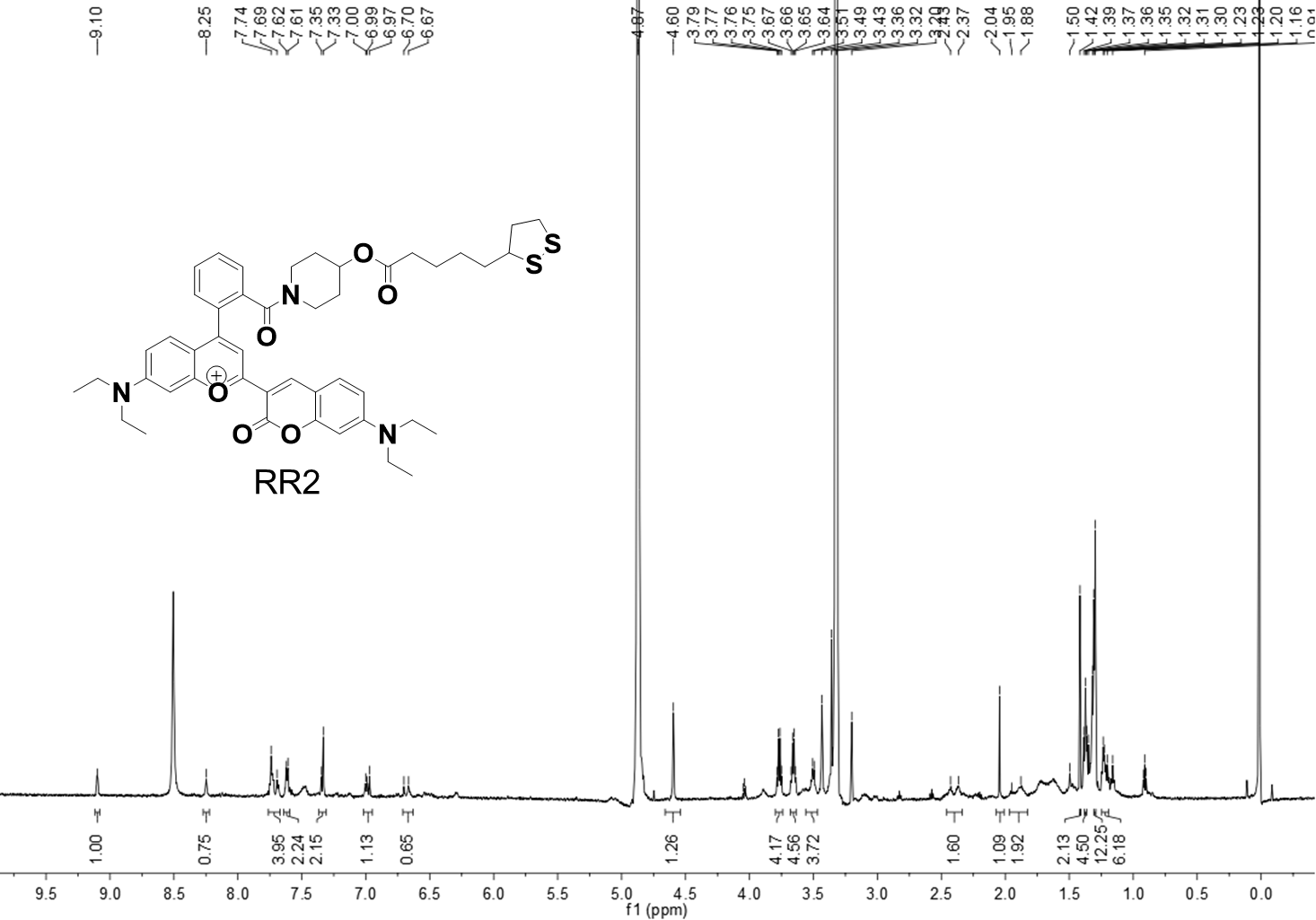
**

**Data S4. ^1^H NMR spectrum of RR2 in MeOD-d4.**

Reference

[1] U. S. Dinish, Z. Song, C. J. H. Ho, G. Balasundaram, A. B. E. Attia, X. Lu, B. Z. Tang, B. Liu, M. Olivo, *Advanced Functional Materials* **2015**, *25*, 2316-2325.

[2] B. Dong, X. Song, X. Kong, C. Wang, Y. Tang, Y. Liu, W. Lin, *Adv Mater* **2016**, *28*, 8755-8759.

[3] F. Neese, *WIREs Computational Molecular Science* **2022**, *12*, e1606.

[4] S. Grimme, A. Hansen, S. Ehlert, J. M. Mewes, *J Chem Phys* **2021**, *154*, 064103.

[5] F. Weigend, R. Ahlrichs, *Phys Chem Chem Phys* **2005**, *7*, 3297-3305.

[6] A. V. Marenich, C. J. Cramer, D. G. Truhlar, *J Phys Chem B* **2009**, *113*, 6378-6396.

[7] aS. Grimme, S. Ehrlich, L. Goerigk, *J Comput Chem* **2011**, *32*, 1456-1465; bS. Grimme, J. Antony, S. Ehrlich, H. Krieg, *J Chem Phys* **2010**, *132*, 154104.

[8] aT. Lu, F. Chen, *J Comput Chem* **2012**, *33*, 580-592; bT. Lu, *J Chem Phys* **2024**, *161*.

[9] R. J. Keizer, M. O. Karlsson, A. Hooker, *CPT Pharmacometrics Syst Pharmacol* **2013**, *2*, e50.
